# Homology-mediated inter-chromosomal interactions in hexaploid wheat lead to specific subgenome territories following polyploidization and introgression

**DOI:** 10.1186/s13059-020-02225-7

**Published:** 2021-01-08

**Authors:** Jizeng Jia, Yilin Xie, Jingfei Cheng, Chuizheng Kong, Meiyue Wang, Lifeng Gao, Fei Zhao, Jingyu Guo, Kai Wang, Guangwei Li, Dangqun Cui, Tiezhu Hu, Guangyao Zhao, Daowen Wang, Zhengang Ru, Yijing Zhang

**Affiliations:** 1 College of Agronomy, State Key Laboratory of Wheat and Maize Crop Science, Henan Agricultural University, 63 Nongye Road, Zhengzhou, 450002 Henan China; 2 Institute of Crop Sciences, Chinese Academy of Agricultural Sciences, Beijing, 100081 China; 3 National Key Laboratory of Plant Molecular Genetics, CAS Center for Excellence in Molecular Plant Sciences, Shanghai Institute of Plant Physiology and Ecology, Shanghai Institutes for Biological Sciences, Chinese Academy of Sciences, 300 Fenglin Road, Shanghai, 200032 China; 4 University of the Chinese Academy of Sciences, Beijing, 100049 China; 5 Henan University, School of Life Science, Kaifeng, 457000 Henan China; 6 Henan Institute of Science and Technology, Eastern Hualan Avenue, Xinxiang City, 453003 Henan Province China; 7 Novogene Co. Ltd, Building 301, Jiuxianqiao North Road, Chaoyang District, Beijing, China

**Keywords:** Wheat, Polyploidization, Introgression, 1RS/1BL translocation, 3D genome, Hi-C, Homology-mediated chromatin interaction, Subgenome-biased TE

## Abstract

**Background:**

Polyploidization and introgression are major events driving plant genome evolution and influencing crop breeding. However, the mechanisms underlying the higher-order chromatin organization of subgenomes and alien chromosomes are largely unknown.

**Results:**

We probe the three-dimensional chromatin architecture of Aikang 58 (AK58), a widely cultivated allohexaploid wheat variety in China carrying the 1RS/1BL translocation chromosome. The regions involved in inter-chromosomal interactions, both within and between subgenomes, have highly similar sequences. Subgenome-specific territories tend to be connected by subgenome-dominant homologous transposable elements (TEs). The alien 1RS chromosomal arm, which was introgressed from rye and differs from its wheat counterpart, has relatively few inter-chromosome interactions with wheat chromosomes. An analysis of local chromatin structures reveals topologically associating domain (TAD)-like regions covering 52% of the AK58 genome, the boundaries of which are enriched with active genes, zinc-finger factor-binding motifs, CHH methylation, and 24-nt small RNAs. The chromatin loops are mostly localized around TAD boundaries, and the number of gene loops is positively associated with gene activity.

**Conclusions:**

The present study reveals the impact of the genetic sequence context on the higher-order chromatin structure and subgenome stability in hexaploid wheat. Specifically, we characterized the sequence homology-mediated inter-chromosome interactions and the non-canonical role of subgenome-biased TEs. Our findings may have profound implications for future investigations of the interplay between genetic sequences and higher-order structures and their consequences on polyploid genome evolution and introgression-based breeding of crop plants.

**Supplementary Information:**

The online version contains supplementary material available at 10.1186/s13059-020-02225-7.

## Background

Chromosomes must be compacted because of limitations in cellular space, but this process must be compatible with biological functional requirements. Common wheat (*Triticum aestivum*; AABBDD, 2n = 6x = 42) is a widely cultivated hexaploid species. Its three subgenomes, which form an extremely large genome (16 Gb) comprising long chromosomes (499–869 Mb), raise a series of interesting questions regarding higher-order chromatin organization and the underlying mechanism. Common wheat is a relatively young hexaploid species that evolved via two polyploidization events [1, 2], the first of which involved a hybridization between the diploid wheat species *Triticum urartu* (AA, 2n = 2x = 14) and an unidentified *Aegilops* species. The resulting tetraploid wheat hybridized with goatgrass (*Aegilops tauschii*; DD, 2n = 2x = 14) to produce an ancestral hexaploid wheat species approximately 10,000 years ago. In previous studies, genomic in situ hybridization (GISH) and Hi-C data revealed that the three subgenomes of hexaploid wheat tend to localize to specific nuclear territories [3–5]. Additionally, interactions are more common between subgenomes A and B than between subgenomes A and D or B and D. These findings indicate that the higher-order chromosomal organization may be maintained following tetraploidization and hexaploidization processes [3]. However, the molecular mechanisms associated with these specific subgenome regions remain largely unknown.

During wheat breeding, chromatin from wild relatives is frequently introduced into domesticated germplasm to promote the development of elite cultivars [6, 7]. The most successful alien introgression for improving wheat disease resistance and yield performance involved the translocation of the short arm of rye (*Secale cereale* L.) chromosome 1R (1RS) to the long arm of wheat chromosome 1B (1BL). The short arm of wheat chromosome 1B (1BS) was replaced by 1RS and resulted in the 1RS/1BL chromosome in the common wheat background. The breeding of the Aikang 58 (AK58) 1RS/1BL line in 2003 represents a major advance in the development of modern commercial wheat varieties in China. This variety has been cultivated on a total of 17 million hectares and has been a major founder parent for wheat breeding in China because of its considerable adaptability to environmental conditions, high yield, and resistance to biotic and abiotic stresses [8]. Examining the interactions of a chromosome carrying alien chromatin in common wheat background is of both theoretical and practical interest.

Local self-interacting domains [i.e., topologically associating domains (TAD) in mammals] are responsible for the main form of intra-chromosomal interactions, which have been proposed to restrict promoter–enhancer interactions; these domains have boundaries that are relatively stable during development [9]. In mammalian cells, the TAD boundaries are generally enriched with euchromatin marks and active genes, indicative of a close relationship between TAD packing and transcriptional regulation [10]. Additionally, mammalian TAD boundaries are typically demarcated by a zinc-finger factor, CCCTC-binding factor (CTCF) [9]. Although its function remains to be conclusively determined, CTCF is believed to serve as an insulator factor that helps maintain the TAD structure [11]. In plants, TAD-like domain structures are uncommon in the most widely analyzed model plant species *Arabidopsis thaliana* (Arabidopsis) [12–15]. In contrast, Hi-C studies on cotton, maize, tomato, sorghum, foxtail millet, and rice revealed TAD-like structures [16–19], the boundaries of which are consistent with high transcriptional activities and active epigenetic architecture. Close orthologs of CTCF genes have not been detected in plants, suggesting plant and animal TAD-like domains may have evolved in parallel [16]. The sequence motif recognized by TCP family transcription factors is reportedly enriched in the boundaries of rice TAD-like domains [16]. Additionally, TCP transcription factor activity was reported to be correlated with 3D structure in *Marchantia* [20]. Considering wheat chromosomes are substantially larger than the chromosomes of model plants, investigating whether novel genetic and epigenetic features exist in wheat chromosome folding domains is warranted.

In mammals, TAD formation can be explained mainly by a “loop extrusion model,” in which cohesins form loops that are stalled at TAD boundaries because of interactions with the boundary CTCF [21, 22]. The anchored ends of the loops share similar epigenetic features and are assumed to be functionally relevant (e.g., distant enhancer–promoter interactions). The expression levels of genes with promoters associated with loops tend to be upregulated [23]. In plants, chromosomal loops have been detected in all species characterized in high-throughput chromatin conformation capture studies, including Arabidopsis [12–15], rice [24], maize [25, 26], and cotton [17], suggesting that they may have conserved biological functions [19]. A recent Hi-C study involving the wheat cultivar Chinese Spring (CS) revealed the highly coordinated expression of loop-connected genes [3]. Therefore, the relationship between chromosomal loops with TADs and their potential regulatory roles in common wheat are interesting issues for further investigation.

We herein characterized the principles of chromatin organization as well as the underlying mechanisms and functional consequences of higher-order contacts, both within and between subgenomes at the inter- and intra-chromosomal levels. Specifically, we analyzed AK58, which is a modern elite cultivar carrying the 1RS/1BL chromosome. The findings provide new insights into the impact of the interplay between the genomic sequence and the higher-order structure on subgenome stability following polyploidizations and introgressions.

## Results

### Influence of mapping stringency on the detection of within-subgenome interactions

**Fig. 1:**
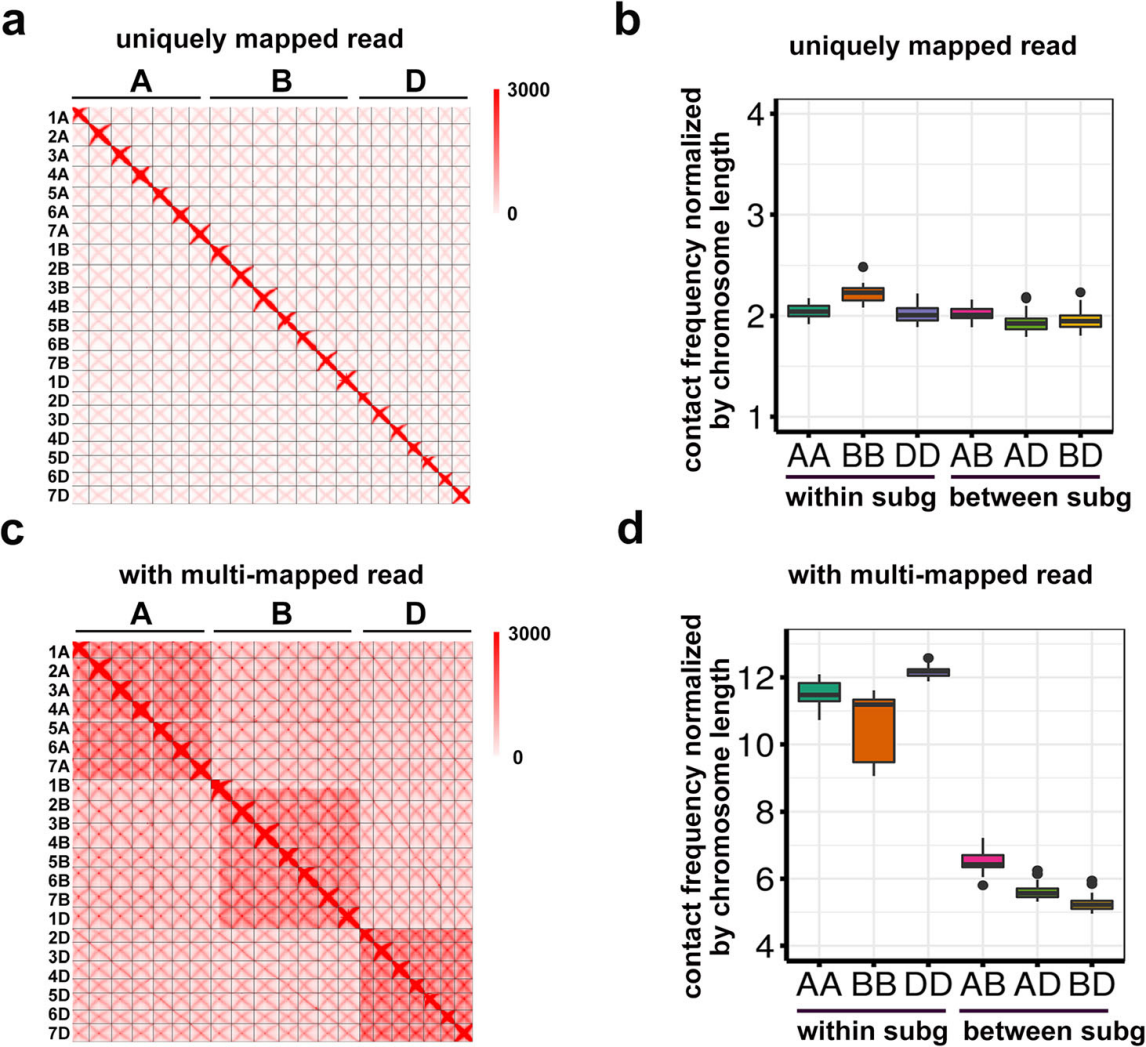
Contact maps and interaction frequencies. **a**, **b** Hi-C contact matrices (**a**) and frequency distributions of all chromosomal interactions (**b**) for all AK58 chromosomes, with only uniquely mapped read pairs used. **c, d** Hi-C contact matrices (**c**) and frequency distributions of all chromosomal interactions (**d**) for all AK58 chromosomes, with both uniquely mapped and multi-mapped read pairs used. "subg" is short for subgenome

We probed whole-genome chromosomal interactions based on an in situ Hi-C analysis. Following the default read-filtering step, we obtained 797.6 million pairs of high-confidence Hi-C reads (data mapping and quality metrics summarized in Additional file 1: Supplemental Table 1). A strong signal was detected along the diagonal of the interaction map, indicative of the considerable abundance of interactions involving nearby genomic regions (Fig. 1a). Interestingly, we also revealed a less prominent anti-diagonal signal that was likely due to the relatively low frequency of connections between the arms of a single chromosome. A similar two diagonal pattern was also reported for barley [27] and reflects the Rabl configuration [28], in which chromosomes fold back with centromeres and telomeres clustering at the opposite sides of the nucleus, leading to the adjacency of long and short arms. These results are consistent with the findings of earlier cytological studies [28, 29] and recent Hi-C report in CS [3].

A recently published three-dimensional map of another wheat landrace cultivar CS based on in situ Hi-C data [3] revealed that within-subgenome chromosomal interactions are considerably more abundant than inter-subgenome interactions. Additionally, subgenome A and B interactions are more frequent than interactions involving subgenome D [3]. The latter observation was confirmed by our data, but the greater frequency of within-subgenome interactions was not obvious from our analysis (Fig. 1a, b). To assess the differences between our analysis and that of the earlier study, we compared each computational step and determined that the major difference was in the mapping stringency. Briefly, our analysis did not allow multiple mapping, whereas the published study [3] included the multi-mapped read pairs. We plotted the interaction map with and without multi-mapped read pairs and observed that the within-subgenome interactions were much more significant when the multi-mapped read pairs were included (Fig. 1c, d and Additional file 1: Supplemental Table 2). Moreover, the interactions between subgenomes A and B also became more prominent. The same results were obtained with published Hi-C data for CS (Additional file 2: Supplemental Fig. 1).

### Subgenome-biased homologous transposable elements (TEs) show high frequency of within-subgenome interactions

**Fig. 2:**
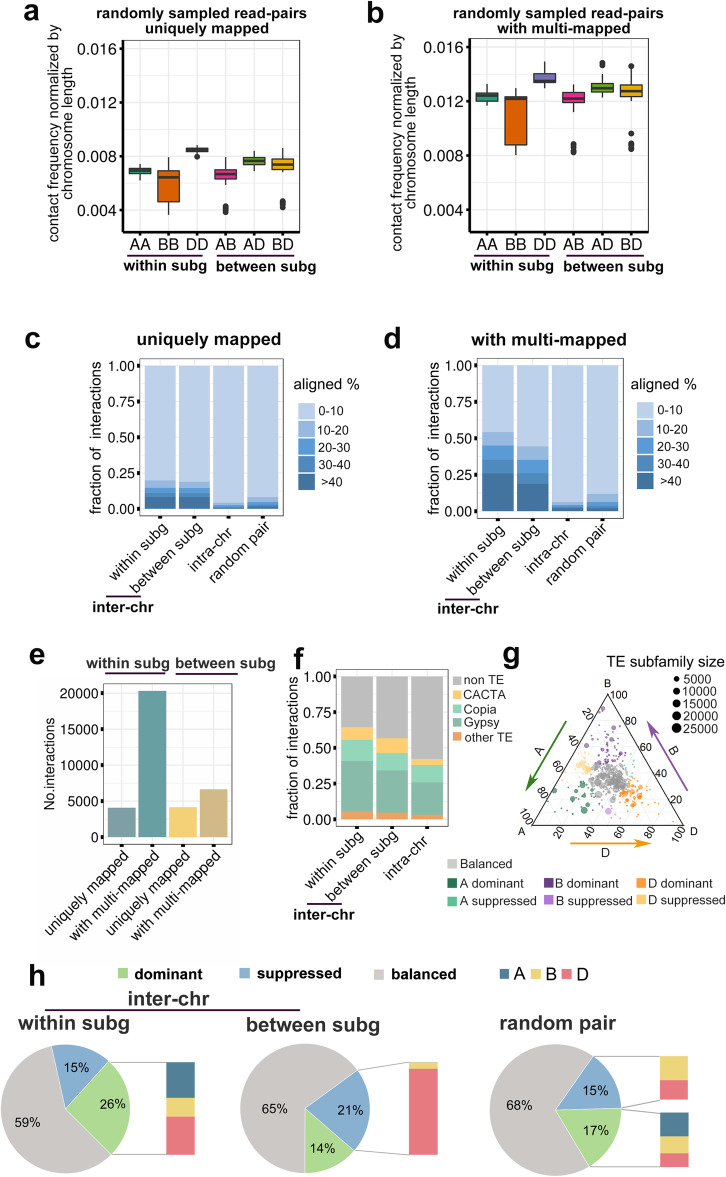
Sequence properties of anchored regions mediating inter-chromosomal interactions. **a, b** For randomly sampled read pairs, the frequency distributions of all chromosomal interactions without (**a**) or with (**b**) multi-mapped reads. Read pairs sampled from CS re-sequencing data [30]. **c, d** Distributions of sequence homology between anchored region pairs of significant inter-chromosomal interactions (inter-chr) within or between subgenomes, as well as anchored region pairs of significant intra-chromosomal interactions (intra-chr). Default BLAST settings were used to compare the sequence homology of read pairs. The proportions of aligned regions are presented. **e** Number of significant inter-chromosomal interactions within or between subgenomes with only uniquely mapped reads or with multi-mapped reads included. **f** Proportions of non-TEs and different types of TEs in the anchored regions supporting different types of chromosomal interactions. Significant inter-chromosome interactions within or between subgenomes were used. **g** Ternary plot presenting the relative abundance of 528 TE families in AK58 following the method published previously [31]. Each circle represents a TE subfamily within subgenome A, B or D; the coordinate represents the abundance of each TE subfamily in each subgenome relative to the abundance of the TE subfamily in all subgenomes. The TE subfamilies close to vertices correspond to single subgenome-dominant categories, whereas the TE subfamilies close to the edges and between vertices correspond to subgenome-suppressed categories. Balanced triads are indicated in gray. **h** Proportions of subgenome-biased TEs in significant inter-chromosomal interacting regions with a high sequence similarity (> 40% of the region alignable between anchored pairs). The distribution of within- and between-subgenome (subg) interacting pairs, as well as the random pairs, is shown

The above mentioned findings prompted us to investigate the biological significance of multi-mapped reads. We designed a randomized test to assess whether multi-mapped read pairs represent background noise. Briefly, paired reads were separately and randomly sampled from the whole-genome re-sequencing data and mapped to the genome with both stringent and non-stringent parameters. Notably, when multi-mapped read pairs were included, the number of interactions within and between subgenomes increased in parallel (Fig. 2a, b). Additionally, experimental evidence from multiple studies supports the existence of subgenome-specific territories [3–5], suggesting the multi-mapped reads may reflect a biological pattern.

We next examined why the inclusion of multi-mapped reads resulted in more prominent inter-chromosomal interactions within subgenomes. Specifically, we were interested in clarifying the type of sequences involved in inter-chromosomal interactions. We speculated that the anchored pairs may share sequence homology. As anticipated, the sequence similarities were significantly higher for the anchored region pairs of inter-chromosomal interactions than for the intra-chromosomal interacting pairs or random pairs (Fig. 2c, d). This indicates that intra- and inter-chromosomal interactions are likely determined by different mechanisms. This result was the same with or without multi-mapped read pairs (Fig. 2c, d). Notably, when multi-mapped reads were included, the number of trans-anchored regions with high sequence homology increased substantially for interactions within subgenomes (Fig. 2e). A further examination of the anchored regions revealed that inter-chromosomal interacting regions within subgenomes have a large proportion of TEs (Fig. 2f). Given the abundant subgenome-biased TEs that evolved independently before polyploidization [32], we postulated that the subgenome-biased homologous TEs may have contributed to the relatively high frequency of interactions within subgenomes. Thus, we defined subgenome-biased TE families based on their relative abundance (Fig. 2g). TEs more abundant in one subgenome are referred to subgenome-dominant TEs, whereas TEs have lower abundance in one subgenome as compared to the other two subgenomes are referred to as subgenome-suppressed TEs. We examined the proportions of subgenome-biased TEs in within-subgenome inter-chromosome interacting regions with high sequence similarity between anchored pairs (> 40% alignable sequence). These anchored pairs were significantly enriched with subgenome-dominant TEs as compared to randomly sampled read pairs  (*P *< 2.2E-16, Fisher's exact test) (Fig. 2h). Accordingly, the substantially greater abundance of inter-chromosomal interactions within-subgenomes is likely due to the extensive exchange of TEs in diploid progenitors. In addition, we observed that the interactions between subgenomes were enriched for subgenome D-suppressed TEs (i.e., higher abundance in subgenomes A and B) (Fig. 2h). Therefore, the more frequent interactions between subgenomes A and B may be ascribed to the accumulated TE exchanges in tetraploid wheat, as reported recently [32]. These results suggest the interactions among the chromosomes of a subgenome are potentially mediated by homologous sequences, and subgenome-dominant homologous TEs likely contribute to the high frequency of chromosomal interactions within subgenomes in hexaploid wheat.

### Low frequency of inter-chromosomal interactions involving the translocated 1RS chromosomal arm

**Fig. 3:**
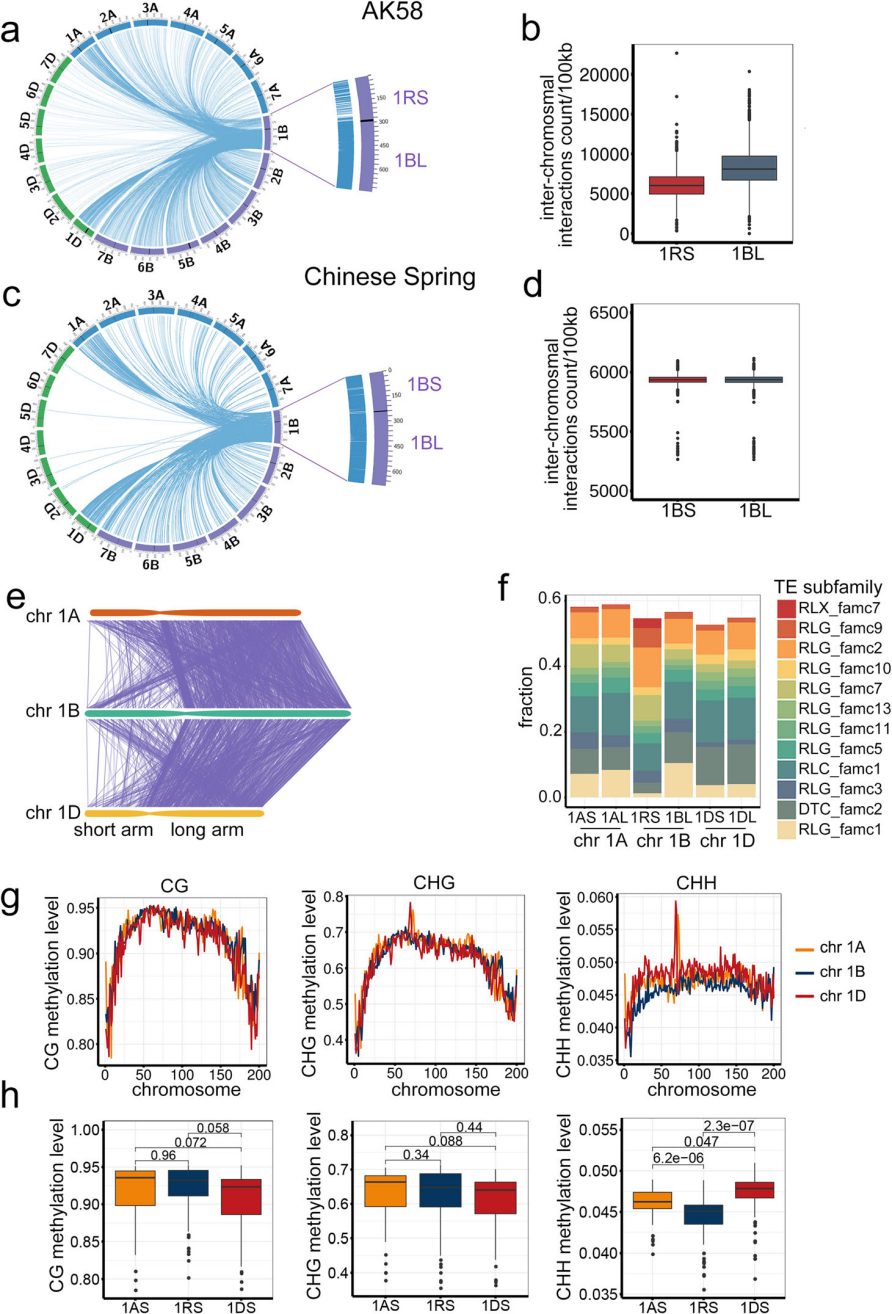
Inter-chromosomal interactions of 1RS/1BL in AK58. **a** Circos plot presenting the inter-chromosomal interactions between chromosome 1RS/1BL and the other chromosomes in AK58. The centromere region is indicated with a black line. Chromosome 1RS/1BL is enlarged (right), with the inter-chromosomal interaction loci of 1RS and 1BL indicated by blue bars. **b** Comparison of the extent of the inter-chromosomal interactions detected for 1RS and 1BL in AK58. The interaction density of 1RS was significantly lower than that of 1BL (*P* < 2.2E−16, Welch’s two-sample *t*-test). **c** Circos plot presenting the inter-chromosomal interactions between chromosome 1B and the other chromosomes in the non-1RS/1BL variety Chinese Spring (CS). **d** Comparison of the extent of the inter-chromosomal interactions detected for 1BS and 1BL in CS (*P* = 0.6014, Welch’s two-sample *t*-test). **e** Synteny plot presenting the sequence homology across chromosomes 1A, 1B, and 1D. Homologous regions are connected by ribbons. **f** Proportion of TE subfamilies in the short and long arms of chromosomes 1A, 1B, and 1D. **g** DNA methylation distribution across chromosomes 1A, 1B, and 1D in three contexts. Each chromosome was divided to 200 consecutive bins, and the DNA methylation level was recorded and plotted for each bin. **h** Box plot showing the DNA methylation levels across 1AS, 1RS, and 1DS. *T*-test was used for pair-wise comparison

Aikang 58 is a wheat–rye 1RS/1BL translocation line. An analysis of the three-dimensional structure revealed that the 1RS region formed a local interacting domain, but had relatively few interactions with other AK58 chromosomes, in contrast to the frequent inter-chromosomal interactions of 1BL (Fig. 3a, b). The same result was obtained with a biological replicate (Additional file 2: Supplemental Fig. 2). In CS wheat, which carries the normal chromosome 1B, similar frequencies of inter-chromosomal interactions were detected for 1BS and 1BL (Fig. 3c, d). These findings are consistent with those of earlier genetic and cytological studies. Alien introgressed fragments in wheat commonly form haploblocks that do not recombine with the recipient genome [33]. Additionally, FISH and GISH results in previous studies indicated that the introgressed rye chromosome fragments tend to form a specific genomic territory [34, 35], possibly because of the relatively low sequence homology between 1RS and wheat chromosomes (Fig. 3e). There is also apparent difference of TE subfamily density between 1RS and the recipient wheat genome (Fig. 3f). We further compared the DNA methylation level across subgenomes. The CG and CHG levels are comparable across chromosomes 1A, 1B, and 1D; however, the CHH methylation is significantly lower in chromosome 1RS (Fig. 3g, h). It would be interesting to know whether there is any relationship between this low CHH methylation and regulation of the introgressed alien chromosome activity. Together, localization in specific genomic territories as well as infrequent inter-chromosomal interactions may represent common higher-order structural features of alien chromatin introgressed into plant genomes.

### Genetic and epigenetic features surrounding TAD-like domains

**Fig. 4:**
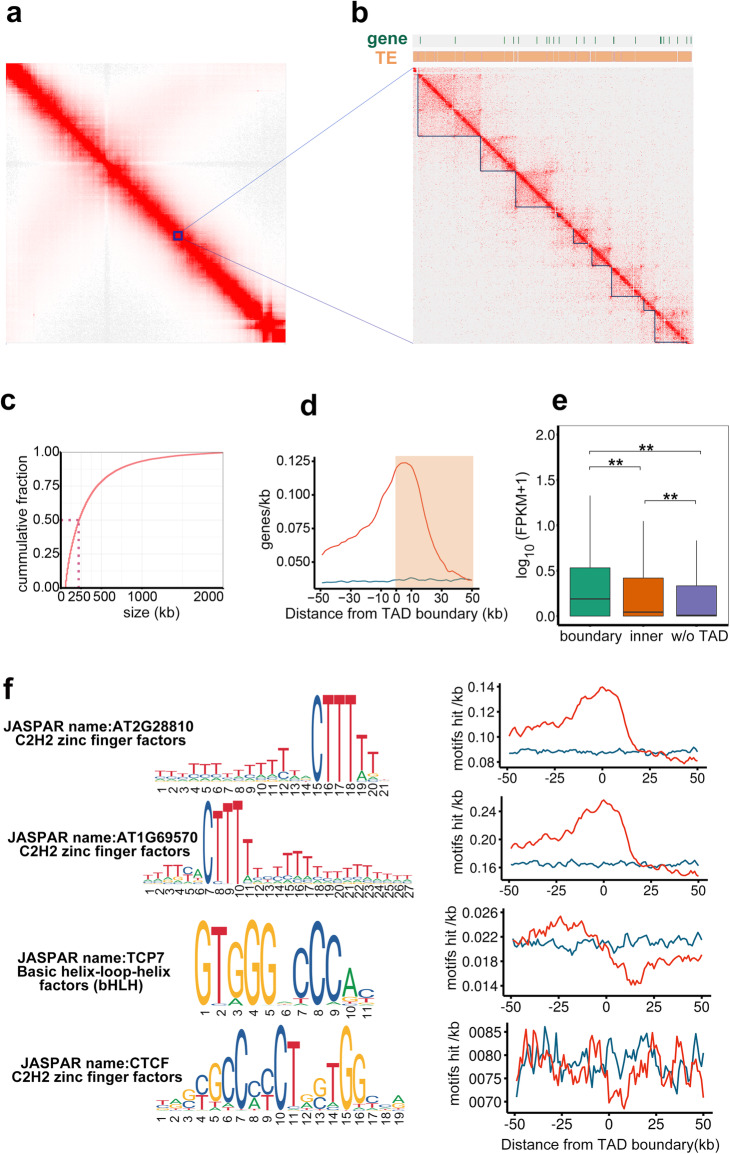
Properties of TAD-like domains in AK58 seedlings. **a** Normalized contact map for chromosome 1A (1-Mb bins). **b** Hi-C contact map for the enlarged region of chromosome 1A (10-kb bins). **c** Cumulative fraction of TAD-like domain sizes in AK58. The *x*-axis presents the TAD length, whereas the *y*-axis presents the fraction of TADs that are shorter than a given length on the *x*-axis. **d** Density of high-confidence gene models surrounding TAD-like domain boundaries. The *y*-axis presents the number of genes per kb. The shaded area indicates the TAD-like domain. Randomly assigned TAD-like domains in genomes were used as the background (blue line). **e** Distributions of gene expression density at the TAD-like domain boundaries, inside the TAD-like domains, and in regions lacking TAD-like domain structures. **f** Sequence motifs enriched at TAD-like domain boundaries. The densities of sequence motifs surrounding TAD-like domain boundaries (per kb) are plotted. Top to bottom: motifs enriched in bread wheat TAD-like domain boundaries (first two panels), TCP motif enriched in rice TAD-like domain boundaries, and the CTCF-binding sequence enriched in human TAD-like domain boundaries. The enrichment statistics are summarized in Additional file 1:Supplemental Table 5

To gain insights regarding local chromosomal organization, we normalized the Hi-C data at a 10-kb resolution, which clarified the patterns of self-interacting domains (i.e., TAD-like domains) (Fig. 4a, b). A total of 21,003 TAD-like domains, covering 52% of the whole genome, were detected (Additional file 1: Supplemental Table 3). The abundance of TAD-like domains was similar across the three subgenomes (Additional file 1: Supplemental Table 4). The median size of common wheat TAD-like domains was approximately 220 kb (Fig. 4c). Moreover, the wheat TAD-like domain boundaries were generally associated with active genes, as previously reported in both plant and human studies [3, 9, 12, 16]. Furthermore, the gene density and gene expression level were both significantly higher at wheat TAD-like domain boundaries than in other genome regions (Fig. 4d, e).

The TAD-like domain boundaries in mammals are typically enriched for zinc-finger factor CTCF [9]. To examine if there are any conserved sequence features in the wheat TAD-like domain boundaries, we performed a de novo motif search followed by an enrichment analysis. Interestingly, A/T-rich motifs were over-represented in the wheat TAD-like domain boundaries. A search of a plant motif database (JASPAR plant) revealed that similar motifs may be recognized by zinc-finger family proteins distantly related to CTCF (Fig. 4f and Additional file 1: Supplemental Table 5).

**Fig. 5:**
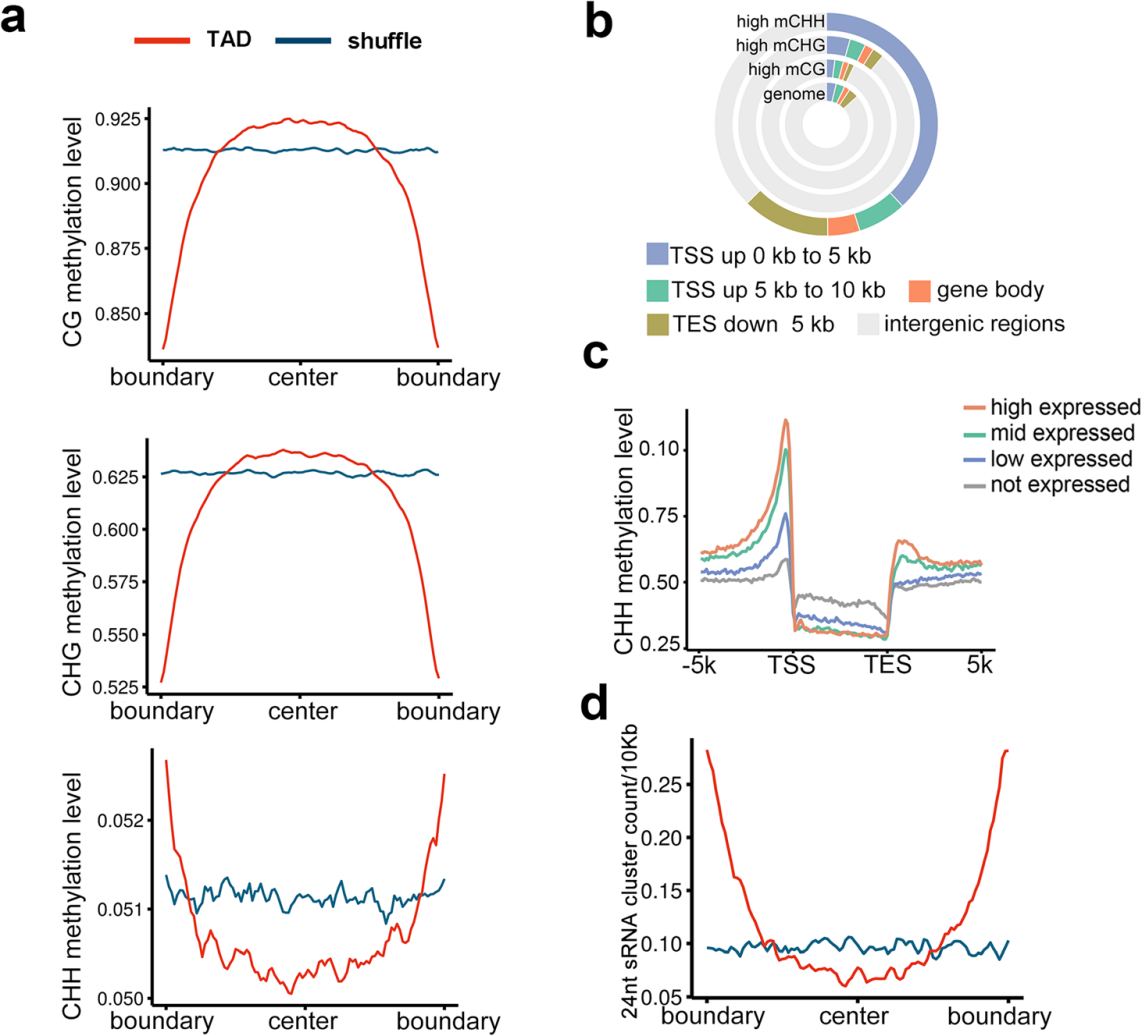
Profiles of DNA methylation and small RNAs at TAD-like domain boundaries. **a** Distributions of DNA methylation in three contexts (i.e., CG, CHG, and CHH) surrounding TAD-like domain boundaries. The background (blue line) was estimated as described for Fig. 4d. **b** Distributions of highly methylated regions in different genomic features. **c** Average CHH methylation surrounding genes grouped by expression levels. TSS, transcription start site; TES, transcription end site. **d** Distributions of 24-nt small RNA sequences surrounding TAD-like domain boundaries. The *y*-axis presents the number of 24-nt sRNA clusters per 10 kb. The background (blue line) was estimated as described for Fig. 4d

Both human and plant TAD-like domains are reportedly associated with specific epigenetic patterns [3, 9, 16]. In the current study, we examined the DNA methylation levels (i.e., mCG, mCHG, and mCHH) surrounding wheat TAD-like domains. Our analysis indicated that the TAD-like domain boundaries lacked CG and CHG methylations (Fig. 5a), consistent with the findings of an earlier investigation of CS [16]. However, the TAD-like domain boundaries were enriched with the CHH methylation. A previous study proved that the CHH methylation is over-represented in sequences surrounding genes in the maize genome, possibly to enforce the boundaries between heterochromatin and euchromatin [36]. Thus, we examined the relationship between the distribution of DNA methylation and gene activities in AK58. The CHH methylation was apparently enriched in the promoter region (Fig. 5b) and was positively associated with gene activity (Fig. 5c). In plants, the CHH methylation is mostly guided by 24-nt small RNA (sRNA) sequences during an epigenetic process unique to plants [37, 38]. Accordingly, we plotted the profiles of 24-nt sRNA sequences surrounding TAD-like domains, which revealed a consistent CHH methylation pattern, with a significant enrichment at the TAD-like domain boundaries (Fig. 5d). Collectively, these results suggest a close association among gene activities, DNA sequence features, DNA methylation, sRNAs, and TAD-like domain formation.

### Regulatory features of chromatin loops

**Fig. 6:**
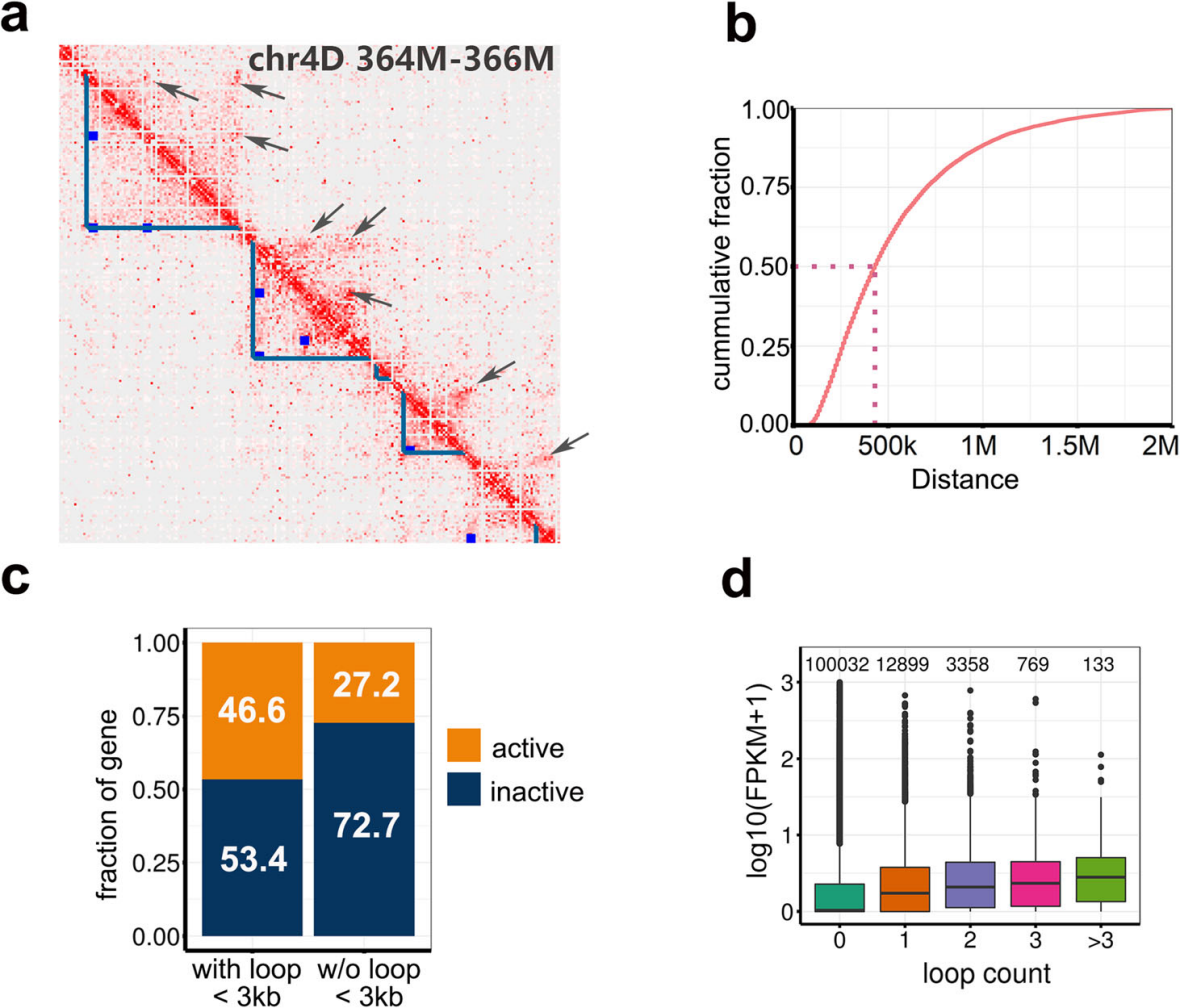
Features of local intra-chromatin loops. **a** Overlap between loops and TAD-like domains. Arrows on the upper diagonal indicate the loops within TAD-like domains or at the boundaries. Blue points on the lower diagonal indicate the positions of the loops corresponding to the arrows. **b** Cumulative fraction of the distance between the anchor points of chromosomal loops. The *x*-axis presents the distance between loop anchors, whereas the *y*-axis presents the fraction of loops that are shorter than a given length on the *x*-axis. **c** Proportions of active and inactive genes located proximally and distally to anchor points. The difference is significant based on Wilcoxon *T* test, *p* < 2e−16. **d** Expression levels of genes varying in the number of interacting loops

The TAD formation in mammals has been ascribed to loop extrusions [21, 22]. We analyzed the positions of chromatin loops (i.e., pairs of regions with a significantly higher frequency of contacts compared with the regions in between). We identified 17,786 pairs of interacting regions, which are hereafter referred to as local anchors (Fig. 6a and Additional file 1: Supplemental Table 6). The median distance between local anchored pairs was approximately 400 kb (Fig. 6b). Moreover, 74.3% of the local anchored pairs were located within or surrounding TAD-like domains, and about half (69%) were localized within gene bodies or promoter regions (3 kb upstream of a transcription start site) (Additional file 2: Supplemental Fig. 3). In a previous human study, loops in promoters were generally associated with enhancers and increased gene activity [23]. Similarly, we observed that genes close to the anchors (< 3 kb) tended to be expressed at significantly higher levels than the genes located further away (Fig. 6c). Furthermore, the gene expression levels tended to increase as the number of loops increased (Fig. 6d). Similar result was also observed using the Hi-C data generated in CS [39] (Additional file 2: Supplemental Fig. 4), possibly because of an increase in the number of distal enhancers.

## Discussion

Previous studies about chromatin 3D map in plants were largely focused on the local chromatin structures. The recent report about inter-chromosomal interactions in wheat was mostly linked to transcriptional regulations [3]. We herein investigated the mechanism affecting higher-order structure on both chromosome and subgenome levels in common wheat. The findings have increased our understanding of the impact of the genetic sequence context on higher-order chromatin structures, which can influence polyploidy stability and plant genome evolution.

### Implications of homology-mediated inter-chromosomal interactions

Stable subgenome inheritance is a fundamental characteristic of polyploid species [40, 41]. Besides the well-known suppression of homoeologous pairing conferred by the *Ph1* locus [42–44], there may be additional mechanisms promoting the increased affinities for wheat chromosomes in the same subgenome. We determined that the high sequence homology associated with subgenome-biased TEs contributes to the high frequency of interactions within subgenomes. In addition to promoting within-subgenome links, homology-mediated inter-chromosome interactions are supported by or can help explain multiple genetic and cytological phenomena at the molecular level. First, the abundant interactions between subgenomes A and B may be due to the substantial genetic communication that developed during the relatively long co-existence period following tetraploidization. Second, the introgressed 1RS fragment has few inter-chromosomal interactions, likely because of the minimal genetic exchange with wheat chromosomes. Third, the Rabl chromosomal configuration has been detected in barley and wheat as well as in other crops with large genomes [28, 29]. An examination of Hi-C matrices indicated that the strong signal along the diagonal is associated with the centromere regions (Fig. 1a, c), implying in wheat cells with Rabl configuration, a major driving force of chromosome folding is likely derived from the centromere. The high density of TEs surrounding centromeres and the inter-chromosomal interactions within subgenomes may facilitate the formation of the Rabl structure. Fourth, the recent finding that inter-chromosomal interactions are more abundant than intra-chromosomal interactions in the autotetraploid species Arabidopsis [45] may be due to a relatively high inter-chromosomal sequence similarity.

### Impact of TEs on higher-order subgenome stability

We revealed a previously uncharacterized role for subgenome-biased TEs related to within-subgenome communication in a hexaploid plant species (wheat). The canonical roles of TEs were previously confirmed to mainly involve changing gene and chromosome structures, developing new regulatory functions, and altering the accompanying epigenetic status [46]. In support of our findings, a recent study in mammals proved that TEs contribute to many loop anchors, some of which help maintain conserved higher-order chromosomal structures [47]. Furthermore, we observed a similar but more prominent pattern in human, where the location of Alu elements, the most proliferative retrotransposon in primates [48], is highly correlated with inter-chromosome interactions (Additional file 2: Supplemental Fig. 5). This is consistent a recent study in metazoan reporting the 3D folding correlated with the clustering of similar repetitive elements [49]. Our findings also raise an interesting question. Previous studies suggested that TEs in polyploid species can mediate inter-element recombinations, ultimately leading to rediploidization [46]. However, our data suggest the subgenome-biased TEs help promote the higher-order subgenome affinity in allohexaploid wheat. Thus, how the chromosomes of a wheat subgenome maintain their structural stability in the face of the high frequency of within-subgenome interactions should be determined. It remains unclear whether there is a mechanism restricting the degree of within-subgenome interactions to balance the maintenance of higher-order chromosomal structures and the sequence stability of the interacting chromosomes.

## Conclusions

Most plant genomes have undergone one or more rounds of polyploidization event, and alien introgressions are often associated with plant genetic improvements. The data presented here provide new insights regarding the sequence homology-mediated inter-chromosome interactions and the non-canonical role of TEs. These findings may have profound implications for future studies on polyploid plant genome evolution and introgression breeding of agriculturally important crops.

## Methods

### Plant materials and growth conditions

This study was completed with bread wheat (*Triticum aestivum*; AABBDD, 2n = 6x = 42) cultivar AK58. Seeds were germinated and cultured in water in Petri dishes for 1 week, after which they were transferred to a nutrient solution. The seedlings were incubated for 2 weeks in a growth chamber with a 16-h light (20 °C): 8-h dark (18 °C) cycle. Leaves were harvested from plants at the 3-leaf stage between 11:00 am and 12:00 pm.

### Bisulfite, RNA, and in situ Hi-C sequencing library preparation and sequencing

Bisulfite and RNA sequencing samples were prepared from 2.2 μg DNA and 2 μg total RNA (rRNA depleted) extracted from the harvested leaves, respectively. For the in situ Hi-C analysis, samples were vacuum-infiltrated with a formaldehyde cross-linking solution. The HindIII restriction enzyme was used to digest the DNA. The digested fragments were ligated with biotin-labeled bases for the subsequent cyclization and capture. After a purification step, the DNA was de-crosslinked and fragmented into 300- to 700-bp sequences to construct the Hi-C libraries. The libraries were constructed and sequenced by Novogene Co. Ltd. (Beijing, China) Specifically, the libraries were sequenced with the HiSeq 3000 system (Illumina) to produce 150-bp paired-end reads. All samples were prepared in biological replicates and displayed high consistency (Additional file 1: Supplemental Table 1 and Additional file 2: Supplemental Fig. 6).

### Hi-C data mapping and contact matrix normalization

A total of 4.3 billion Hi-C read pairs were aligned to the AK58 genome and filtered by HiC-Pro (version 2.11.1) [50]. All relevant statistics are summarized in Additional file 1: Supplemental Table 1. Briefly, a two-step filtering process was used to ensure the chimeric reads were accurately aligned. After the reads were mapped, the low-quality reads and singletons were discarded. The aligned read pairs were assigned to HindIII restriction fragments, and the invalid pairs with an overhanging end or those that self-ligated or re-ligated were discarded. The default parameter “-q 10” was used to retain unique mapped read pairs, and was changed to “-m” to retain multi-mapped pairs. Finally, 797.6 million uniquely mapped and 1546.8 million multi-mapped pairs were obtained. A Hi-C replicated dataset was generated with DpnII, and all of the main presented data were reproduced in the replicate (Additional file 2: Supplemental Fig. 2).

The genome was divided into 10-kb bins, and the number of contacts between each bin pair was recorded to construct an original contact map. The number of contacts between loci *i* and *j* was denoted as *M*_*ij*_. Because of the variance in chromatin accessibility, nucleosome occupancy, comparability, and restriction enzyme site density during the preparation of the Hi-C libraries, the original interaction matrix needed to be normalized (methods reviewed in [23]). The vanilla coverage (VC) method has been widely used to normalize Hi-C data. Briefly, each element in the matrix was divided by the sum of the respective row and subsequently divided by the sum of the respective column. We applied the Knight and Ruiz (KR) normalization algorithm, which is a modified VC method corrected for all factors that may cause biases without explicit modeling. The algorithm involves a repeated VC normalization of *M*_*ij*_ until the sums of all of the rows and columns are the same value [51].

### Detection of TAD-like domains and loops

We used “findTADsAndLoops.pl” implemented in the Homer software to simultaneously detect TAD-like domains and loops [52]. First, the normalized Hi-C interaction matrices with overlapping windows were generated, after which each chromosome was scanned for triangle domains or locally dense regions of contacts that have a high degree of inter-domain interactions relative to their surrounding regions. The loops were scored based on their Hi-C interaction density, whereas the TAD-like domains were scored according to an inclusion ratio, which represented the ratio of within-TAD interactions to TAD interactions with the surrounding region (i.e., regions upstream and downstream of the TAD that are the same size as the TAD). Default settings were applied to consider only interactions between regions within 2 Mb of each another. The loops ≥ 3× the window size were considered. Candidate loops were those that satisfied the following criteria: interaction count > 1.5-fold greater than the local average interaction count (5× the window size) and > 2-fold greater than the global average interaction count for regions with the same interaction distance along the chromosome.

To determine the resolution for the subsequent analysis, we examined the fragment size distribution following the digestion (Additional file 2: Supplemental Fig. 7). The data indicated that 96.9% of the fragments were shorter than 10 kb, which was chosen as the resolution for detecting TADs and loops. The loops were validated by the recently published 3C-PCR results that were used to test the Hi-C data for CS wheat [3]. All four loops had co-linear regions in AK58, and three were also detected as loops in AK58, whereas the fourth was localized to the TAD boundaries in AK58 (Additional file 2: Supplemental Fig. 8).

We generated KR-normalized contact matrices with bin sizes set to 2.5 M, 1 M, 500 K, 250 K, 100 K, 50 K, 25 K, 10 K, and 5 K with Juicer and visualized the TADs with Juicerbox [53]. The TAD-like domains were clearly distinguished at a 10-kb resolution (Additional file 2: Supplemental Fig. 9). Additionally, data for the TAD-like domain boundaries and loops were highly consistent at 10 kb resolution.

### Detection of significant inter-chromosomal interactions

We used Juicer to obtain the normalized contact frequency [53], whereas “INTER_KR” was applied to normalize the inter-chromosomal interaction frequency at a 10-kb resolution. To eliminate the effects of chromosomal translocations, we removed the regions that interacted with multiple consecutive sites on other chromosomes. To avoid enlarging the low-coverage regions after the standardization, only contacts among the top 10% of raw contacts and the top 10% of normalized contacts were considered as significant interactions and selected for the subsequent analysis.

### Analysis of Chinese Spring Hi-C data

We downloaded the Hi-C data of Chinese Spring from the NCBI BioProject database (accession number GSE133885) [54]. Reads were aligned to the International Wheat Genome Sequencing Consortium reference sequence (version 1.0). The data analysis procedures are the same with the analysis for Hi-C data in AK58. For the detection of intra-chromosomal loops, since the depth of this data is lower than AK58, the parameter of “findTADsAndLoops.pl” were adjusted to “-poissonLoopLocalBg 0.05; poissonLoopGlobalBg 0.05” to reach a comparable number of loops with AK58. For the detection of significant inter-chromosomal interactions, the same methods as AK58 were used.

### RNA sequencing data analysis

Sequencing reads were cleaned as described above. The HISAT2 program (version 2.1.0) [55] was used for mapping the RNA sequencing reads to the reference AK58 genome sequence and gene models. The gene expression levels were calculated as the number of fragments per kilobase of transcript per million mapped reads (FPKM) to account for the gene length and sequencing depth. Small RNA sequencing reads were trimmed with Trimmomatic (version 0.36) [39] to remove Illumina adapters and low-quality bases (quality score < 20). The ShortStack program (version 3.85) was used to map the 24-nt clean reads and detect small RNA clusters [56]. The clusters with high read density (number of read within the cluster per million mapped read > 1) were used for analysis.

### Bisulfite sequencing data analysis

Sequencing reads were cleaned with the Trim Galore (version 0.4.4), Trimmomatic (version 0.36) [39], and Sickle programs, which eliminated bases with low-quality scores (< 25), irregular GC contents, sequencing adapters, and short reads. The remaining clean reads were then aligned to the AK58 genome with the default settings of the Bismark program (version 0.19.0) [57]. The default settings were strict, with only the best unique alignments reported, and all non-unique alignments were removed [57]. Thus, we applied only two additional filtering steps, namely the removal of reads with a mapping quality < 20, followed by the removal of PCR duplicates with deduplicate_bismark implemented in Bismark. The extent of the cytosine methylation was determined with bismark_methylation_extractor implemented in Bismark. Next, the cytosine methylation ratio was calculated as the number of mCs divided by the number of reads covering the position. Bases covered by fewer than three reads were considered low-confidence positions, and their methylation ratios were not recorded.

### Detection of subgenome-dominant and subgenome-repressed TEs

We used CLARI-TE to annotate AK58 TEs as previously described for the annotation of TEs in CS [32]. Additionally, ClariTeRep is a library containing the TEs and repeated sequences annotated in the TREP database and the annotated repeats on CS chromosome 3B [58]. Along with this library, RepeatMasker [59] was used to scan the whole genome to detect candidate TEs. The results were prepared in an “embl” format to be used as the input file for CLARI-TE, which revealed the TE types, genomic positions, families, and subfamilies. The TE families were designated according to the rules of the ClariTeRep database. For example, DTC_famc2.1 and DTC_famc2.2 are subfamilies of DTC_famc2.

To standardize the relative abundance of each TE family across subgenomes, for each chromosome, the lengths of the TEs belonging to one TE family were summed and divided by the length of a given chromosome. The relative abundance of each TE family was calculated as the sum of the normalized lengths of the TEs belonging to the same family on all chromosomes. To detect the subgenome-biased TE subfamilies, a previously described ternary plot-based method was applied [31]. Briefly, the relative abundance of each TE family present in one subgenome was divided by the total relative abundance of the given TE family in all three subgenomes. The relative contributions of each subgenome per subfamily were used to plot the ternary diagrams. We calculated the Euclidean distance between the relative position of each subfamily and each of the seven ideal categories (i.e., subgenome A-, B-, or D-dominant, subgenome A-, B-, or D-suppressed, and TE families with a similar abundance across subgenomes).

### Motif analysis

The motifs at the TAD-like domain boundaries were analyzed with MEME-ChIP [60]. A 5-kb region flanking the TAD-like domain boundary was chosen as the primary sequence input, whereas the internal TAD-like domain sequence was chosen as a control. The analysis was completed with the parameter “-meme-maxw 12 -meme-nmotifs 6 -meme-p 12 -ccut 0”. The motifs were then scanned against the regions around TAD-like regions using the Find Individual Motif Occurrences program of the MEME software toolkit [61].

### Supplementary Information

**Additional file 1.** Supplemental Tables S1-S6.

**Additional file 2.** Supplemental Figure S1-S9.

**Additional file 3.** Review history.

## Data Availability

The datasets generated during the current study, including Hi-C data, bisulfite sequencing data, as well as RNA sequencing data are available in the Gene Expression Omnibus (GEO) repository https://www.ncbi.nlm.nih.gov/geo/ under accession number GSE139020 [54]. Other published data sets used include Chinese Spring Hi-C data [63] and human Hi-C data [62].
