# Author Correction: Homology-mediated inter-chromosomal interactions in hexaploid wheat lead to specific subgenome territories following polyploidization and introgression

**DOI:** 10.1186/s13059-023-03154-x

**Published:** 2024-01-02

**Authors:** Jizeng Jia, Yilin Xie, Jingfei Cheng, Chuizheng Kong, Meiyue Wang, Lifeng Gao, Fei Zhao, Jingyu Guo, Kai Wang, Guangwei Li, Dangqun Cui, Tiezhu Hu, Guangyao Zhao, Daowen Wang, Zhengang Ru, Yijing Zhang

**Affiliations:** 1 College of Agronomy, State Key Laboratory of Wheat and Maize Crop Science, Henan Agricultural University, 63 Nongye Road, Zhengzhou, 450002 Henan China; 2 Institute of Crop Sciences, Chinese Academy of Agricultural Sciences, Beijing, 100081 China; 3 National Key Laboratory of Plant Molecular Genetics, CAS Center for Excellence in Molecular Plant Sciences, Shanghai Institute of Plant Physiology and Ecology, Shanghai Institutes for Biological Sciences, Chinese Academy of Sciences, 300 Fenglin Road, Shanghai, 200032 China; 4 University of the Chinese Academy of Sciences, Beijing, 100049 China; 5 School of Life Science, Henan University, Kaifeng, 457000 Henan China; 6 Henan Institute of Science and Technology, Eastern Hualan Avenue, Xinxiang City, 453003 Henan Province China; 7 Novogene Co. Ltd, Building 301, Jiuxianqiao North Road, Beijing, Chaoyang District China

**Correction: Genome Biol 22, 26 (2021)**

**https://doi.org/10.1186/s13059-020-02225-7**

Following publication of the original article [1], the authors indicated the following three errors:

1. The corresponding authors (Yijing Zhang) has changed institutions and wanted to change her contact email to zhangyijing@fudan.edu.cn; 495591339@qq.com
2. The following information was omitted in the **Availability of data and materials** section.
  The genome sequence data reported in this paper have been deposited in the Genome Warehouse at the China National Center for Bioinformation under accession number GWHBFXN00000000 and are publicly accessible at https://ngdc.cncb.ac.cn/gwh/Assembly/23313/show.
  An updated genome version is released with the recent Molecular Plant paper (10.1016/j.molp.2023.10.015), and is available at https://ngdc.cncb.ac.cn/gwh/Assembly/9670/show under the accession number GWHANRF00000000. A comprehensive AK58 genome database is available at https://triticeae.henau.edu.cn/aikang58/, providing the updated genome sequence, genome annotations and JBrowse viewer of SNPs, QTLs, and multiple epi-modifications.
3. The authors would like to include some additional analyses they have done (Additional file 4). This includes the Word document that contains text and a figure, as well as the code/scripts attached.

### Supplementary Information

**Additional file 4.** Additional analyses.
