# Supplementary material for: Homology-mediated inter-chromosomal interactions in hexaploid wheat lead to specific subgenome territories following polyploidization and introgression
Source: Genome Biol. 2021 Jan 8;22:26. doi: 10.1186/s13059-020-02225-7 (PMC7792079; doi:10.1186/s13059-020-02225-7)
Supplement: Supplementary file 2 — Additional file 2. Supplemental Figure S1-S9. [file 13059_2020_2225_MOESM2_ESM.docx]

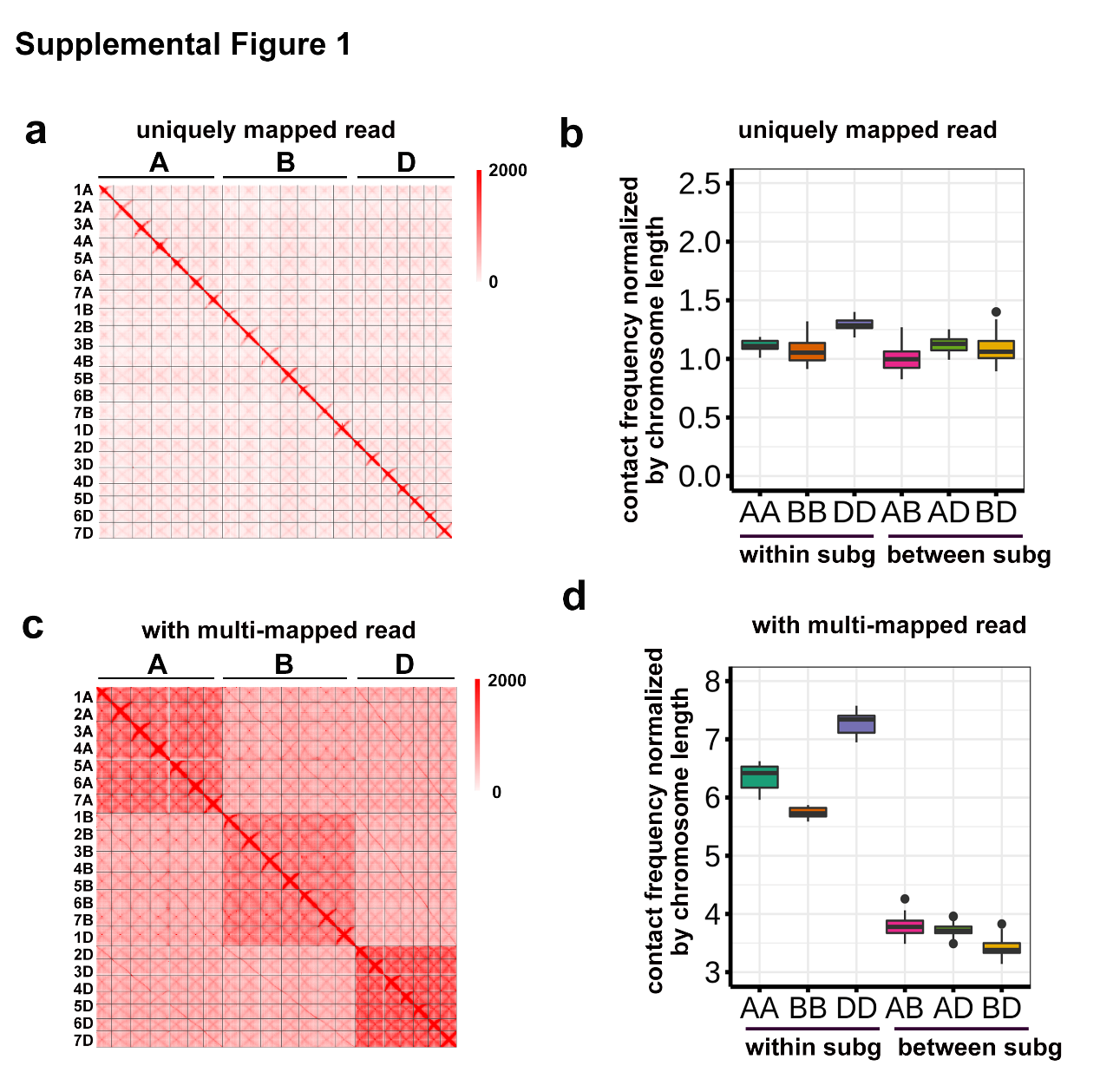


**Supplemental Figure 1. Contact maps and interaction frequencies using data from Chinese Spring (GSE133885).**

**a, b.** Hi-C contact matrices (a) and frequency distributions of chromosomal interactions (b) for all chromosomes, with only uniquely mapped read pairs used.

**c, d.** Hi-C contact matrices (c) and frequency distributions of chromosomal interactions (d) for all chromosomes, with uniquely mapped and multi-mapped read pairs used.


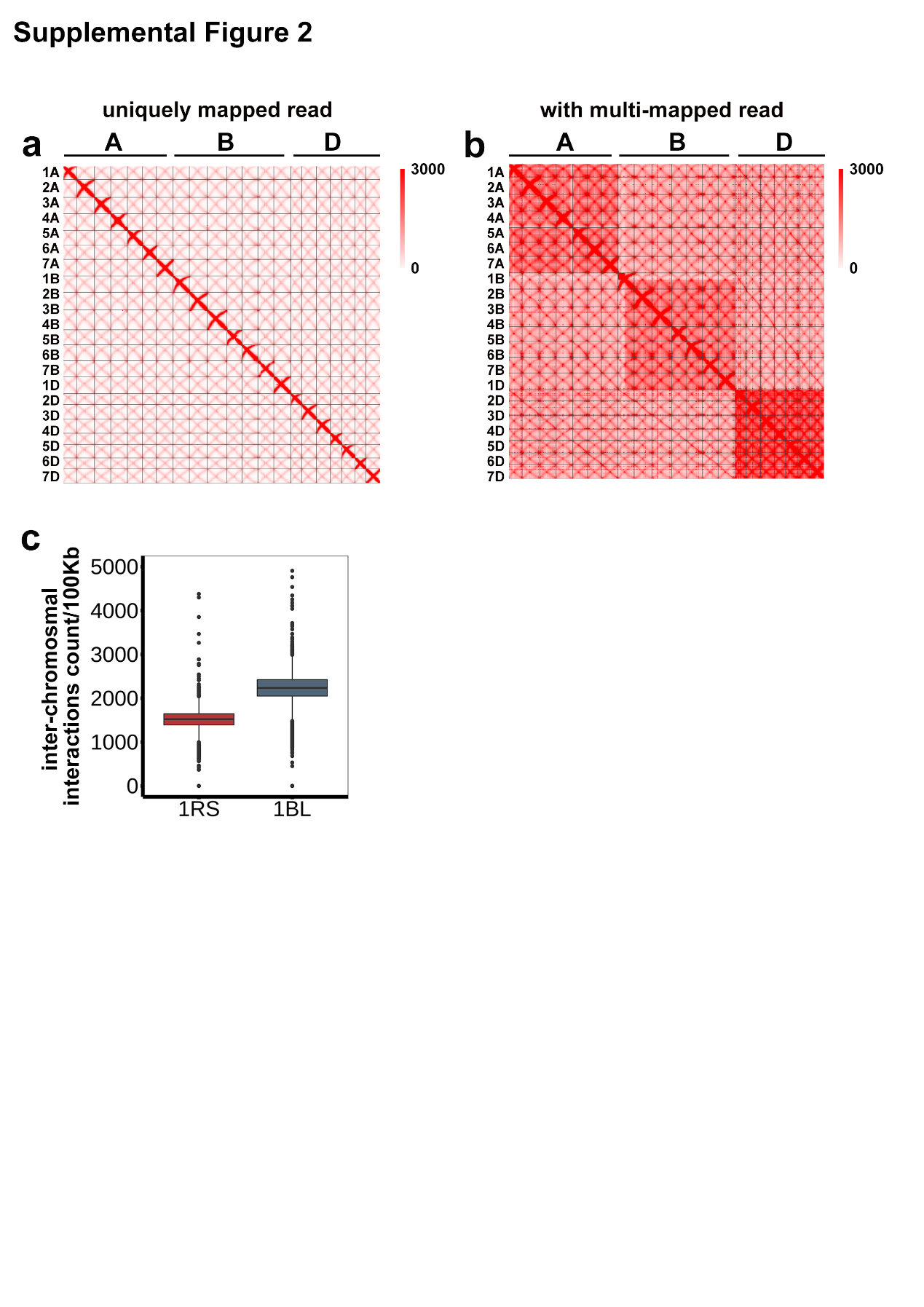


**Supplemental Figure 2. The major conclusions validated by Hi-C biological replicate using DpnII as enzyme.**

1. Hi-C contact matrices with only uniquely mapped read pairs used.
2. Hi-C contact matrices including multi-mapped read pairs.
3. Comparison of the extent of the inter-chromosomal interactions detected for 1RS and 1BL using Hi-C replicate of AK58.

**
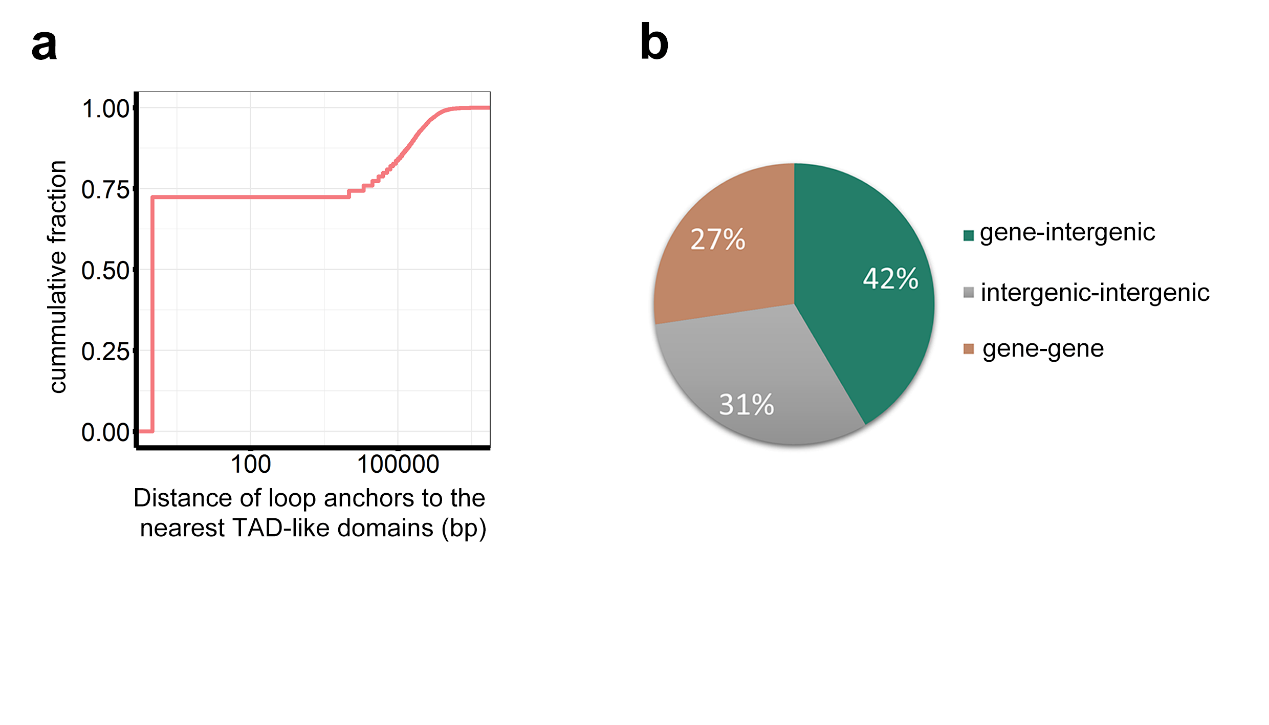
**

**Supplemental Figure 3. Distribution of local anchored pairs surrounding TADs and genes.**

1. Cumulative fraction of the distance of loops anchors to the nearest TAD-like domains.
2. Proportion of different combinations of genomic regions at loop anchor loci.


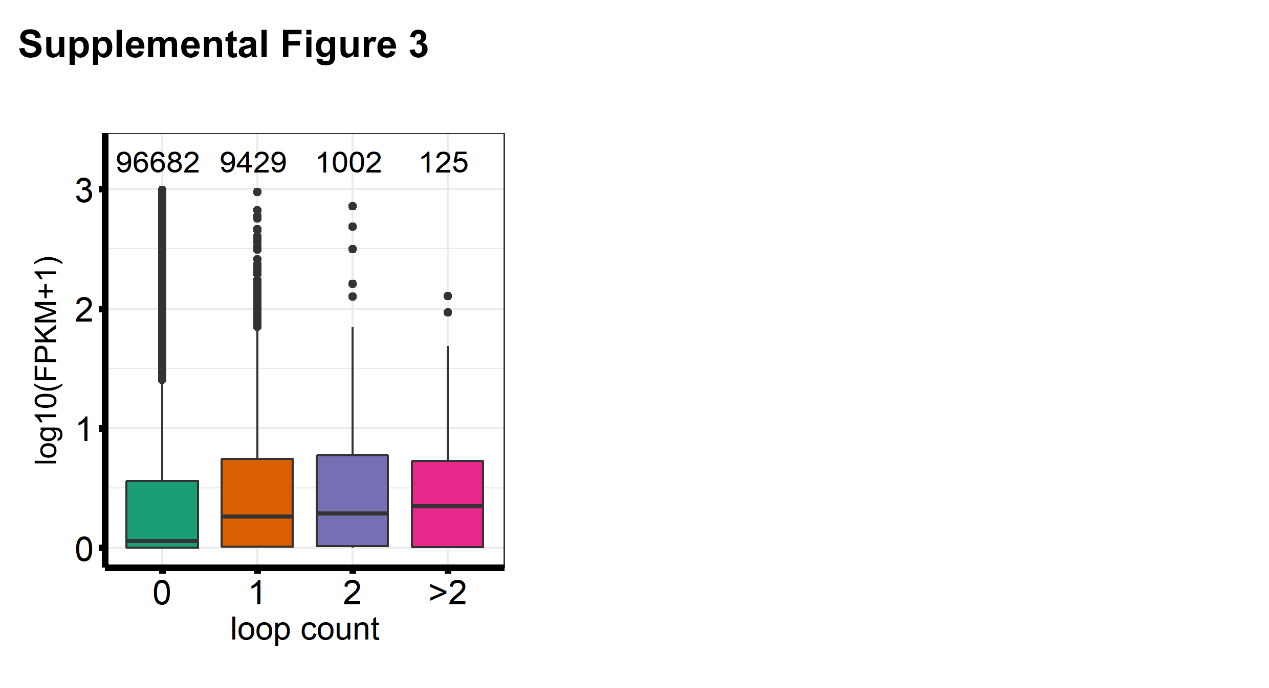


**Supplemental Figure 4. Expression levels of genes varying in the number of interacting loops. Data from Chinese Spring (GSE133885).**


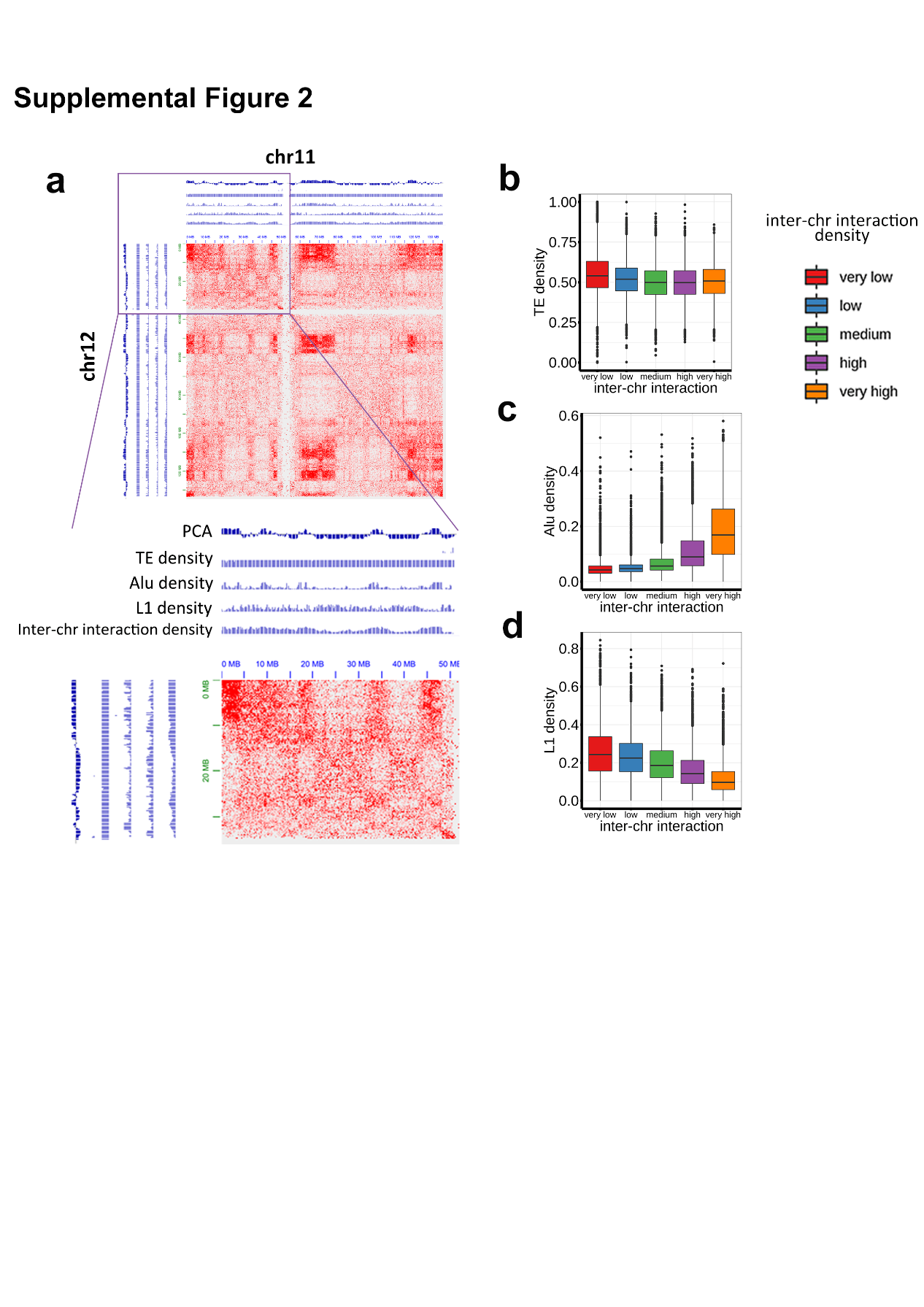


**Supplemental Figure 5. The association between Alu density and inter-chromosomal interactions in Homo sapiens (GSE63525).**

1. Hi-C contact matrices of Homo sapiens between chromosome 11 and 12, as well as the densities of TE, Alu and L1 type TE in human.

**b, c, d.** Densities of total TE (b), Alu (c) and L1 (d) with different levels of inter-chromosomal interactions


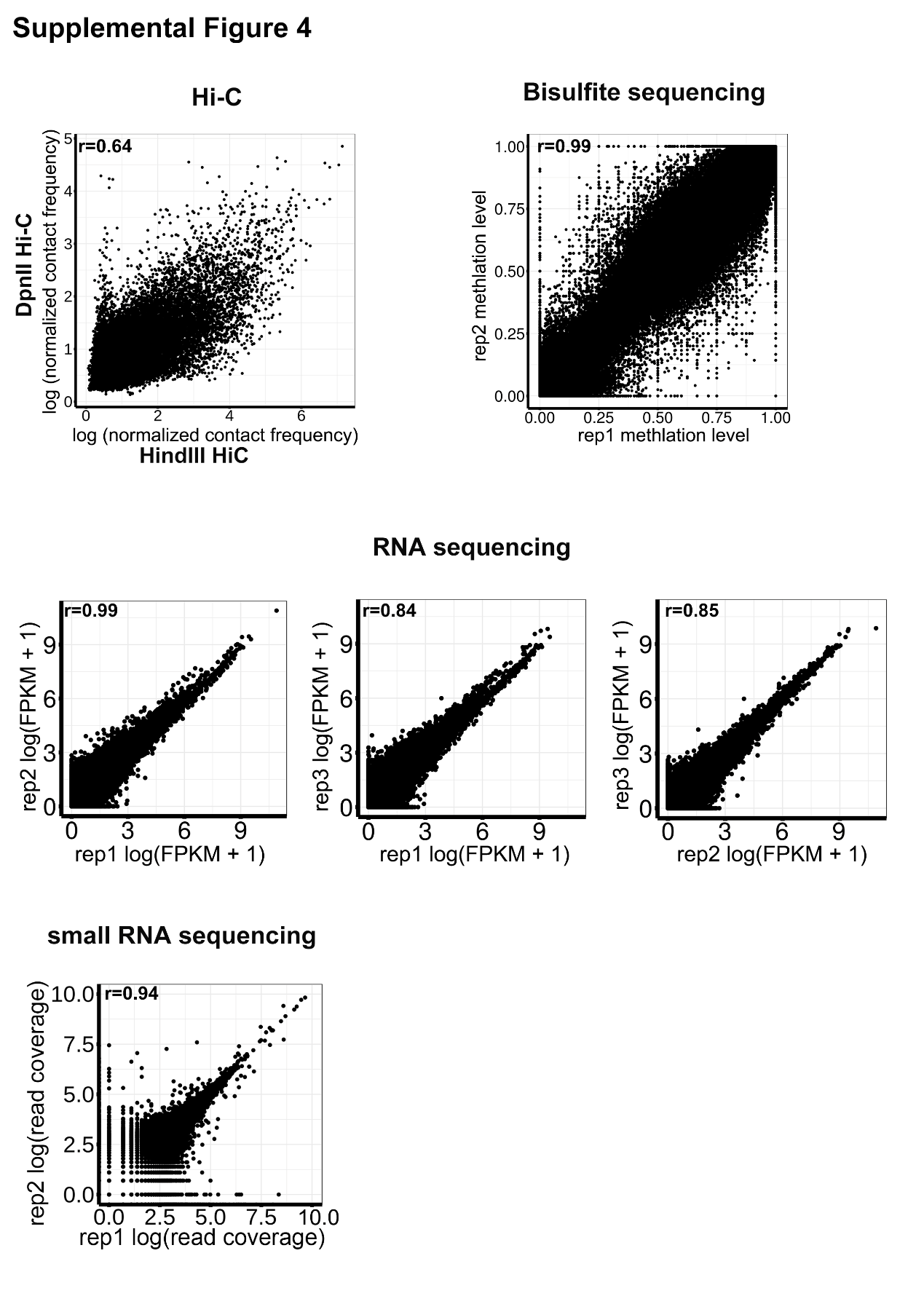


**Supplemental Figure 6. Scatter plots showing the correlation between replicates.** For Hi-C dataset, the number of read pairs within each 50 kb bin was calculated. For BS-seq data, the level of methylated C was recorded. For RNA-seq data, expression level measured by FPKM for all genes were compared.


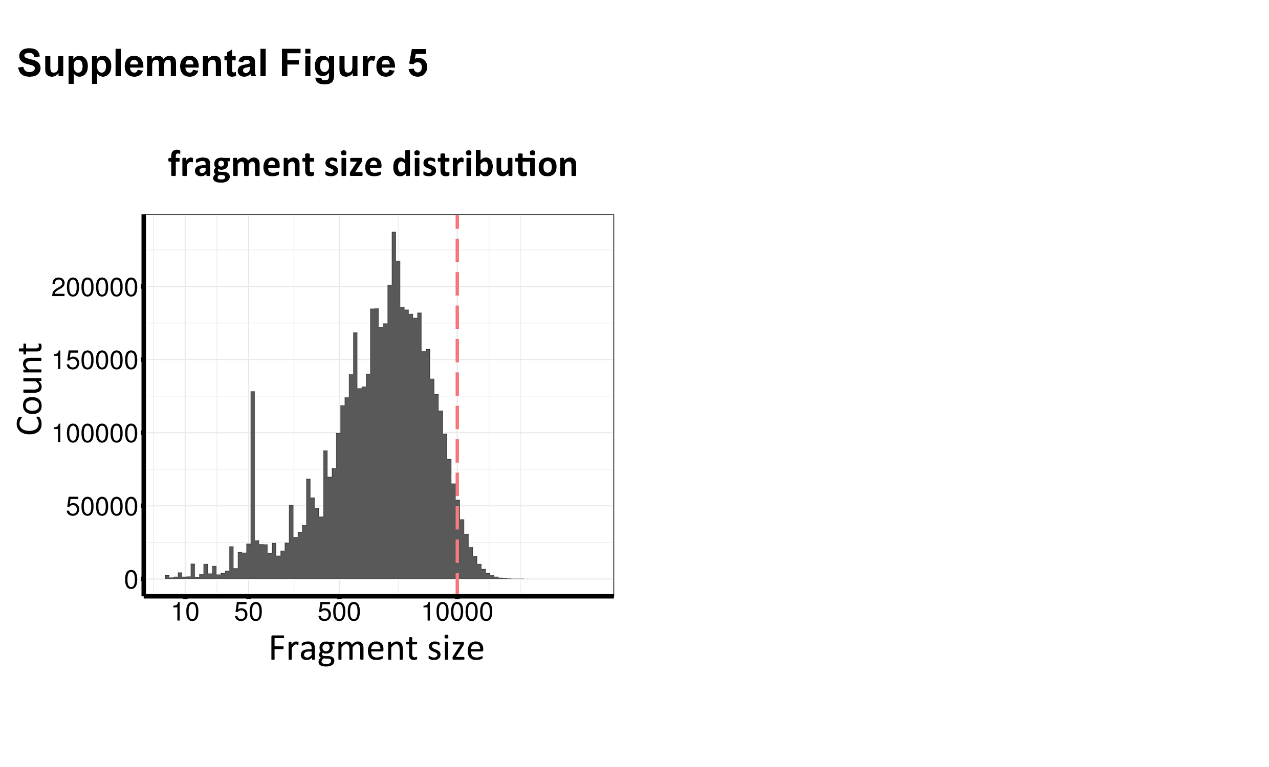
­­**Supplemental Figure 7. Size distribution of the digested fragment.**


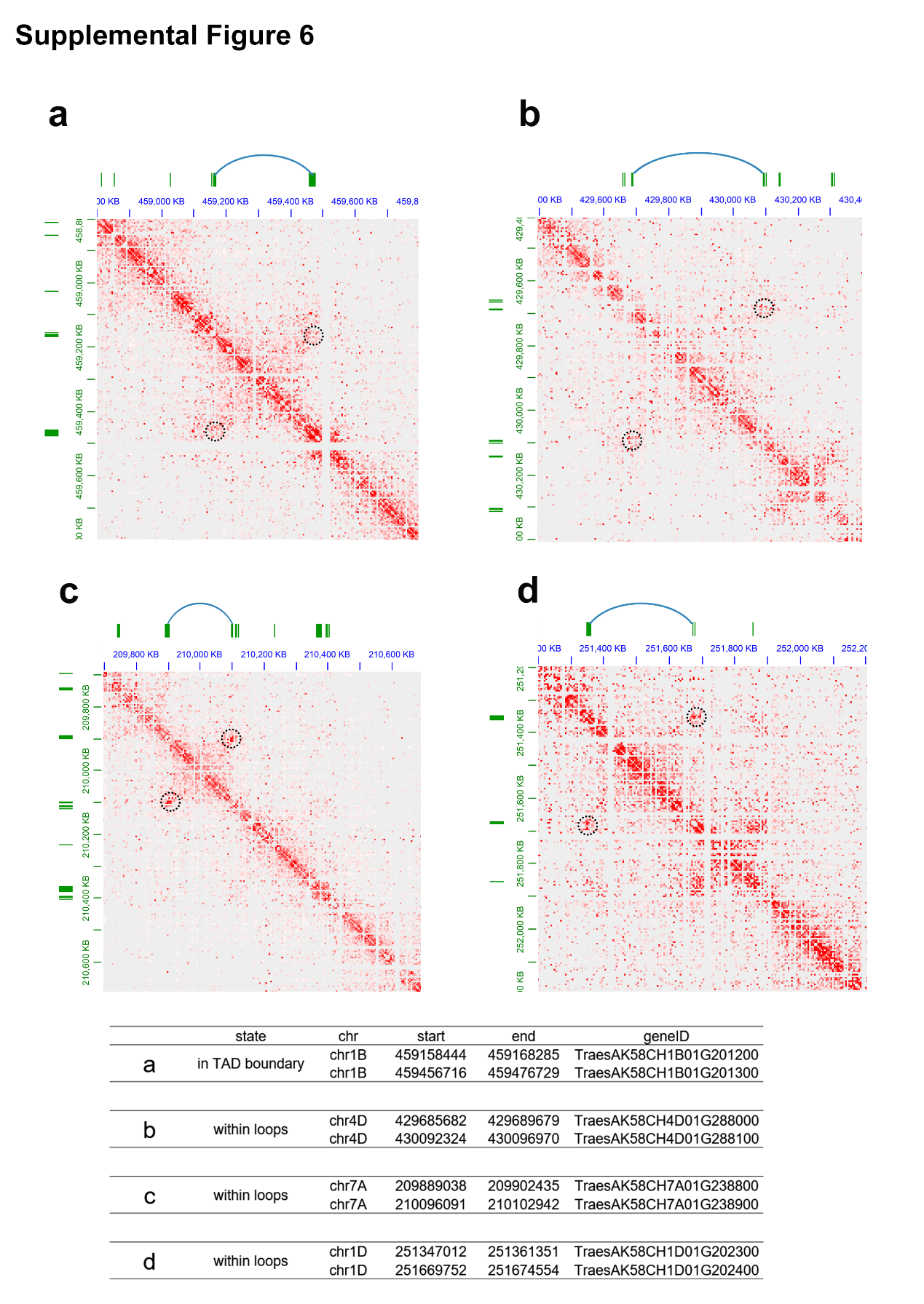


**Supplemental Figure 8. Validation of detected loops by the 3C-PCR.**


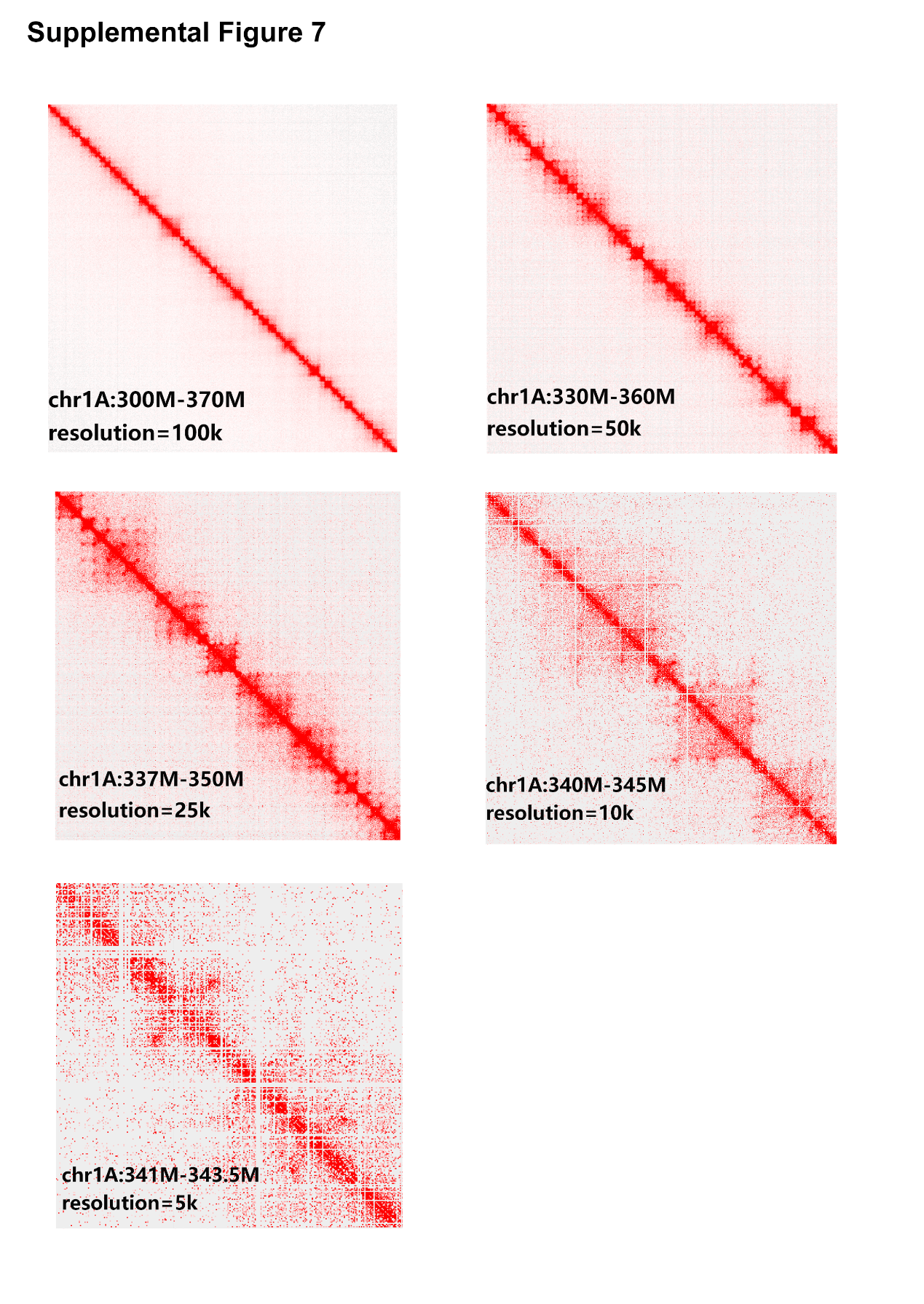


**Supplemental Figure 9. Interaction maps at different resolutions.**
