# Supplementary material for: Homology-mediated inter-chromosomal interactions in hexaploid wheat lead to specific subgenome territories following polyploidization and introgression
Source: Genome Biol. 2021 Jan 8;22:26. doi: 10.1186/s13059-020-02225-7 (PMC7792079; doi:10.1186/s13059-020-02225-7)
Supplement: Supplementary file 3 — Additional file 3. Review history. [file 13059_2020_2225_MOESM3_ESM.docx]

Review History

**First round of review**

**Reviewer 1**

**Are you able to assess all statistics in the manuscript, including the appropriateness of statistical tests used?** No, I do not feel adequately qualified to assess the statistics.

**Comments to author:**

The manuscript by Jia et al describes the chromatin architecture of bread wheat, an important polyploid species in agriculture. In particular the cultivar used, AK58, contains the well-known 1BL/1RS wheat-rye chromosome translocation. This choice allows the authors to examine how a recently acquired chromosomal domain interacts with the rest of the genome. The study includes interesting findings on the interactions between chromosomes belonging to the same and to different sub-genomes, and with the introgressed chromosome arm 1RS. At a local scale, long-range folding domains, called "TADs" in the text, are identified. More precisely, the authors report the identification of 25,431 topologically associated domains (TADs) covering 49% of AK58 genome. The presence of active genes, zinc-finger binding sequences, 24-nt small RNAs and low DNA methylation levels are shown at the boundaries of such structures, suggesting a scenario partially similar to those observed in Metazoans. Interestingly, authors observed also that interactions between subgenomes A and B were significantly more frequent as compared to their interactions with the more recently introduced subgenome D. In line with this, Jia et al., also found limited inter-chromosomal interactions with the alien (translocated) chromosomal region 1RS, as compared to the dense 24 inter-chromosomal interactions detected for 1BL
While providing useful and promising insights into wheat chromatin conformation, the study suffers from major technical and editorial flaws that need to be addressed (see below). In addition, authors should put more effort in the Introduction, Discussion and Methods sections, which are more than minimal and superficial.

Major issues:
1)     The manuscript does not include any experimental validation of Hi-C results. Albeit the Methods section is elusive, I understand that only one replicate has been used for both Hi-C and RNA-seq experiments, which is problematic. For example, low frequency of inter-chromosome interactions detected for 16 translocated 1RS chromosome arm is interesting and has to be confirmed. More generally, all main observation should be confirmed using an independent replicate and a complementary methodology. For instance, DNA FISH or GISH is required to demonstrate the consistency of the reports (in particular to confirm the interesting observations made on inter-chromosome and inter-subgenome interactions (as in Martin et al., 2018 Front. Plant Sci., 2018).
2)     The authors do not provide a data quality summary about Hi-C data, such as those provided by HiC-Pro statistics, as common in the field. To evaluate if the datasets is valid it is necessary provide a table with the number of total reads, mapping %, number of dangling ends, singleton, valid pairs, etc… Same issue with the for RNA-Seq and bisulfite sequencing.
3)     Authors did not detail sufficiently how they defined TADs. Also, they state that "TADs could be distinguished at 10k" but how did-they define this 10K window as being the best suitable resolution? Same issue for the detection of loops: these are critical analyses in this study. Therefore at least a few loops must be confirmed by either DNA FISH or 3C-qPCR analysis of specific loci.
4)     Same issue for motif detection. Authors did not show any P-value, any metrics. In this context it is impossible to assess the validity of the reports.
5)     Same issue for the DNA methylation analyses. Nothing is described about the number of reads, genome coverage, the quality of bisulfite conversion. In this context again, the validity of the experiments performed can hardly be assessed.
6)     The authors do not provide a data quality summary about Hi-C data, such as those provided by HiC-Pro statistics, as common in the field. To evaluate if the datasets is valid it is necessary provide a table with the number of total reads, mapping %, number of dangling ends, singleton, valid pairs, etc… Same issue with the for RNA-Seq and bisulfite sequencing.
7)     The analysis of Hi-C data contains severe inconsistencies (see below) especially regarding the achieved resolution, that appears to be unrealistic given the size and repeat content of wheat genome.  As indicated on page 9, the enzyme used to generate Hi-C data in this study is HindIII, a six-base cutter. Therefore, the theoretical resolution limit correspond to its average cutting frequency of 4-kb ( [1/4]6 = 1/4096 bp ). However, the authors claim they can distinguish anchors of loops within or farther than 3 kb of TSS of genes, which would be an extremely high resolution even for small and non-repetitive genomes. Moreover, the resolution used to find loops with HOMER is 5-kb (Methods, page 15 line 12: "-res 5000 -window 25000"). Can the authors reconcile these inconsistencies?
8)     Editorial issues: the Methods section is very approximative, making it difficult to reproduce the results and/or to compare them across species and cultivars. The Discussion section is extremely short and very superficial as a simple summary of the observations, without providing any model or hypothesis explaining them. This section should contain at least a comparison with other plant species that highlight differences and similarities. The language is frequently imprecise and are present many typographical errors, sometimes making the reading difficult (see minor issues below). Also, the labeling of the figures is often unclear (see below) and the English should be proofread.

Specific comments:
    Main text:
-     Page 2, line 13: "TADs" is the acronym of "Topologically associating domains" not "Topologically associated domains". This mistake is present throughout the manuscript. Moreover, given the still-debated nature of folding domains in plants, it would be more appropriate to use the term "TAD-like" rather than "TADs".
-     Page 4, line 21: Reference numbering after 11 is shifted and need to be fixed. In addition, reference 11 is not related to TADs.
-     Page 4, line 21: It would be appropriate to cite the study of Dong et al. (https://doi.org/10.1111/jipb.12809 )
-     Page 5, line 6-7: In addition of genome size, I would mention the chromosome size (500-800 Mbp) that is several times larger than maize and rice and may influence the folding domains.
-     Page 6, line 6-9: the origin of the introgression (rye) should me mentioned.
-     Page 7, line 3: "stringent read filtering" is a very generic term in this context, since the stringency of pairs filtering cannot be modified in HiC-Pro. However, the stringency of mapping can be tuned. If non-default setting were used, they should be fully described in the Method section. If not, "stringent" need to be deleted.
-     Page 7, line 4: "map of genome packing" is commonly referred as "interaction map"
-     Page 7, line 17: "with 10 kb bins" should be "at 10-kb resolution".
-     Page 7, line 17: It should be clarified that the lack of "bias" refers to absence of differences of TADs density between the three sub-genomes.
-     Page 8, line 2: Reference 4 is related to bacteria and not relevant here.
-     Page 10, line 1: Is the word "significantly" supported by any statistical test ?
-     Page 10, line 18-21 and figure 5 a-b: The boxplot show the number of significant interactions, not the density of interactions (normalized by chromosome size and number). Does this calculation take in account A) the different chromosome size and B) the different number of possible interactions for each type (21 intra-chromosomal, 21 inter-chromosomal between homeologous, 63 within each sub-genome and 126 inter-chromosomal between different genomes) ?  
-     The resolution of 5-kb for inter-chromosomal interactions seems unrealistic at this sequencing depth (Methods, page 15 line 16: "-res 5000 -window 25000 -minDist 10000000"). Moreover, a minimum distance of 10 Mega-bases does not exclude long-range intra-chromosomal interaction, considering the chromosome size in wheat is between 500 and 800 Mbp.
-     Page 12 line 11-15: The existence of homology-mediated inter-chromosomal interactions is a major finding and should be placed among results rather than in Discussion. A finer analysis that takes in account the interactions between different sub-genomes, similarly to what done for Figure 5 c, would better support the hypothesis that the increased interactions between subgenomes A and B (Figure 5) is driven by sequence homology.
-     Page 17 line 20: Gene expression is measured as "RPM". To compare different genes within a given sample, it is necessary to normalize for the transcripts length (i.e. TPM or RPKM), as longer transcripts will generate more reads. Do RPM account for this factor? Which pipeline/software was used to analyze RNA-Seq data?

Figures and legends:
-     Legend of figure 1c: "fractions of genes" is unclear. Does this refer to average gene coverage? How the coverage was calculated? Sliding window, non-overlapping bins? In either case, of which size?
-     Legend of Figure 1d: when referring to the density of genomic features (genes, TEs, smRNAs, etc…) the unit of measure should be specified on the Y-axis (%, genes/Mb, etc..)
-     Legend of Figure 1e: "Distribution of expression density of genes" is very unclear. Is it possibly "distribution of gene expression levels"? I suggest to use a linear scale instead of log2 transformed values and/or exclude the outliers to improve the readability of this chart.
-     Page 8, line 10: "Fig 2" and "Fig 3" are mislabeled throughout the text. According to the order in which they are mentioned in the text, Figure 3, that shows motifs, should go before figure 2, that illustrates DNA methylation.
-     Legend of Figure 4d: scale on the X-axis is missing.
-     The interaction intensity between for different type of chromosomes is measured as the number of significant interactions (Figure 5a-b), while the interaction intensity between different sub-genomes (Figure 5 c), between the introgressed chromosome arm 1RS and the rest of the genome (Figure 6 b) is measured as interaction density. What is the reason for using different measures? Is it possible to use a single measure for all the interaction intensities?
-     Similarly, in Figure 5 a-b is plotted the number of significant interactions, but the level of sequence homology in Supplemental Figure 2 uses all pairwise interactions. Why different sets of pairwise interactions are used? How would the plots look by using the same set of interactions?

**Reviewer 2**

**Are you able to assess all statistics in the manuscript, including the appropriateness of statistical tests used?** There are no statistics in the manuscript.

**Comments to author:**

The authors selected the wheat variety AK58 in the study and this is an ideal system to do 3D genome mapping not only for homologous and homeologous chromosomes, but also for alien chromosome and native chromatin. However, the manuscript is basically descriptive, and current presentation of the results is insufficient. I can't find new conclusions from this version of paper compared to many published plants 3D genome mapping studies.

Major concerns:

1. It seems that all sequencing libraries and primary sequencing data of Hi-C, RNA-seq and BS-seq in this study were prepared and generated by a third-party profit company Novogene. What I am really concerned is the data quality controls, which were not described in the manuscript including the replicate information, reproducibility between replicates if they have. In addition, the ratio of valid reads in Hi-C assay is too low that thus reduce the reliability of conclusions based on it.

2. They identified tens of thousands of TADs and similar number of chromatin loops, however, they observed that the median size of common TADs (~200 kb) is smaller than the loops (400 kb). We usually consider that chromatin loops are kind of sub-TADs features and have the shorter genomic spans.

3. They described in the main text that they try to detect the chromatin loops following the method of Eres's 2014 Cell paper (Rao et al). However, they stated in the Method section that the TADs and Loops were called using a suite of Homer software "findTADsAndLoops.pl" published in 2010. I am confused with the inconsistent analysis methods.

4. The authors characterized the epigenomic features of TAD boundaries using DNA methylation data, but they failed to provide any novel conclusion. In addition, the same group had published a bunch of wheat histone modifications data (Li et al., Genome Biology 2019). I would suggest the authors take a look at the epigenomic features of TAD boundaries with these datasets.

5. I suggest the authors to do some validations for detected high-frequent interacting anchors using FISH or 3C methods.

6.  The Discussion is too perfunctory since many plant 3D genome studies had been published and the authors did not cite any of them in this part of the manuscript.

7. This manuscript was prepared in a hurry and a lot of writing issues including incorrect citation (e.g. reference #11 in p4-line21), Font inconsistence (e.g. p14-line2) and format issues (e.g. p16-line14) could be easily found throughout the paragraphs.

**Authors Response**

We sincerely thank the reviewers for the cogent comments to help us improve our manuscript, which we have addressed in detail below. We apologize for the haste of the last version of manuscript, which we carefully revised according to all the comments.

Before the one-by-one response, we need to mention that there is a wheat Hi-C data published using wheat cultivar Chinese Spring (doi.org/10.1186/s13059-020-01998-1) during our revision process. We compared our results with the published one and observed one obvious difference. In the published study, one important discovery is that there are much more abundant interactions among chromosomes from the same subgenome (Following Figure a), which has also been validated by GISH. This pattern was not obvious from our Hi-C result (Following Figure b). Since both our study and the published study adopted the same experimental procedure (in-situ Hi-C) and computational tools, we compared each computational step, and determined that the major difference was in the mapping stringency. Briefly, our analysis did not allow multiple mapping, whereas the published study included the multi-mapped read pairs. We plotted the interaction map with and without multi-mapped read pairs and observed that the within-subgenome interactions were much more significant when the multi-mapped read pairs were included (Following Figure c-d). The same results were obtained with published Hi-C data for CS (Following Figure e-f).Through random sampling strategy, we demonstrated that the number of interactions within and between subgenomes increased in parallel for randomly sampled read pairs (Following Figure g-h), and it is most likely that the signal presented by multi-mapped reads may reflect a biological pattern. We further examined the anchored regions mediating within subgenome interactions and revealed that subgenome biased homologous TEs contributed to the high frequency of within subgenome interactions. Please refer to the first two parts of Results section (pages 8-11) and Figures 1-2 for details.

*Genome Biology* 21, 104 (2020)

Our result

Data from *Genome Biology* 21, 104 (2020)

Random sampled data

Our data

Here are one-by-one responses to reviewers.

**Reviewer1：**

**1)** The manuscript does not include any experimental validation of Hi-C results. Albeit the Methods section is elusive, I understand that only one replicate has been used for both Hi-C and RNA-seq experiments, which is problematic. For example, low frequency of inter-chromosome interactions detected for 16 translocated 1RS chromosome arm is interesting and has to be confirmed. More generally, all main observation should be confirmed using an independent replicate and a complementary methodology. For instance, DNA FISH or GISH is required to demonstrate the consistency of the reports (in particular to confirm the interesting observations made on inter-chromosome and inter-subgenome interactions (as in Martin et al., 2018 Front. Plant Sci., 2018).

**Response:** Thank you for pointing this out. In the last version, we have one Hi-C dataset, two RNA-seq replicates, two DNA methylation replicates and two small RNA-seq replicates. In the revised manuscript, we added one Hi-C replicate a different enzyme for digestion. All replicates displayed high consistency (please refer to Supplemental Fig. 4 and page 18, line 12-14 in Methods section). All main presented results based on Hi-C data using HindIII as enzyme for digestion were reproduced in the replicate using DpnII as enzyme (please refer to Supplemental Fig. 2, and page 19, line 1-2). Data summary and statistics are listed in Supplemental Table 1.

In addition, there are other experimental evidence supporting the conclusions which we referred to and discussed in the manuscript:

1. The conclusion of more frequent interactions between A and B as compared to their interactions with D was also observed by the published Hi-C data from Chinese Spring (page 5, line 16-17 and page 9, line 1-2).
2. The conclusion of reduced interaction with 1RS is supported by the published 3D-FISH and FISH results, which are described here and are also referenced in page 12, line 3-5.
(a) Recent 3D-FISH result revealed the introgressed rye chromosomes occupied discrete positions in wheat nucleus (Instability of Alien Chromosome Introgressions in Wheat Associated with Improper Positioning in the Nucleus. Int J Mol Sci 2019）.

**rye DNA**

**Nucleus with a pair of rye del1RS.1RL chromosomes in wheat background after 3D-FISH.**

Yellow: rye DNA

Magenta: centromeres

Green: telomere-specific sequence

Blue: all nuclear DNA

White dashed lines: nucleoli areas

(b) In another study, it was revealed that in a wheat-rye chromosome substitution line, the rye chromosomes exist as independent domains and occupy discrete territories in the interphase nuclei. ( Non-Rabl Patterns of Centromere and Telomere Distribution in the Interphase Nuclei of Plant Cells. Chromosome Research 1998, 6, 551-558)

**An interphase nucleus from a wheat-rye 2R-2D chromosome substitution line**

Blue: nuclei DNA

Red: rye DNA

Yellow: telomeric DNA

Green: centromeric DNA

Red: telomeric DNA

**2)**  The authors do not provide a data quality summary about Hi-C data, such as those provided by HiC-Pro statistics, as common in the field. To evaluate if the datasets is valid it is necessary provide a table with the number of total reads, mapping %, number of dangling ends, singleton, valid pairs, etc… Same issue with the for RNA-Seq and bisulfite sequencing.

**Response:** We provided the data summary and statistics in Supplemental Table 1 for all sequencing data including Hi-C, BS-seq, RNA-seq and smRNA-seq.

**3)** Authors did not detail sufficiently how they defined TADs. Also, they state that "TADs could be distinguished at 10k" but how did-they define this 10K window as being the best suitable resolution? Same issue for the detection of loops: these are critical analyses in this study. Therefore at least a few loops must be confirmed by either DNA FISH or 3C-qPCR analysis of specific loci.

**Response:** We re-wrote the method section and added all necessary details (please refer to the Method section).

To determine the resolution for Hi-C data analysis, we examined the fragment size distribution following the digestion (Supplemental Fig. 5). The data indicated that 96.9% of the fragments were shorter than 10 kb, which was chosen as the resolution for detecting TADs and loops (please refer to page 20, line 12-15).

The loops were validated by the recently published 3C-PCR results that were used to test the Hi-C data for CS wheat (doi.org/10.1186/s13059-020-01998-1). All four CS loops had co-linear regions in AK58, and three were also detected as loops in AK58, whereas the fourth was localized to the TAD boundaries in AK58 (Supplemental Fig. 6)(please also refer to page 20, line 15-19).

**4)** The analysis of Hi-C data contains severe inconsistencies (see below) especially regarding the achieved resolution, that appears to be unrealistic given the size and repeat content of wheat genome. As indicated on page 9, the enzyme used to generate Hi-C data in this study is HindIII, a six-base cutter. Therefore, the theoretical resolution limit correspond to its average cutting frequency of 4-kb ( [1/4]6 = 1/4096 bp ). However, the authors claim they can distinguish anchors of loops within or farther than 3 kb of TSS of genes, which would be an extremely high resolution even for small and non-repetitive genomes. Moreover, the resolution used to find loops with HOMER is 5-kb (Methods, page 15 line 12: "-res 5000 -window 25000"). Can the authors reconcile these inconsistencies?

**Response:** Thank you for the suggestion. We used 10-kb resolution for all Hi-C data analysis as described in the response to the major comment 3.

The enrichment of 10 kb loop anchors within 3 kb of TSS is the calculated from the average profile (the following is a schematic).

**5)**	Same issue for motif detection. Authors did not show any P-value, any metrics. In this context it is impossible to assess the validity of the reports.

**Response:** We added a new Supplementary table 5 listed all the enriched motifs enriched surrounding TAD boundaries, as well as the metrices.

**6)**	Same issue for the DNA methylation analyses. Nothing is described about the number of reads, genome coverage, the quality of bisulfite conversion. In this context again, the validity of the experiments performed can hardly be assessed.

**Response:** We provided the data summary and statistics in Supplemental Table 1 for all sequencing data including Hi-C, BS-seq, RNA-seq and smRNA-seq.

**7)**	Editorial issues: the Methods section is very approximative, making it difficult to reproduce the results and/or to compare them across species and cultivars. The Discussion section is extremely short and very superficial as a simple summary of the observations, without providing any model or hypothesis explaining them. This section should contain at least a comparison with other plant species that highlight differences and similarities. The language is frequently imprecise and are present many typographical errors, sometimes making the reading difficult (see minor issues below). Also, the labeling of the figures is often unclear (see below) and the English should be proofread.

**Response:** We carefully re-wrote the manuscript and added necessary details.

**Specific comments:**
Main text:
- Page 2, line 13: "TADs" is the acronym of "Topologically associating domains" not "Topologically associated domains". This mistake is present throughout the manuscript. Moreover, given the still-debated nature of folding domains in plants, it would be more appropriate to use the term "TAD-like" rather than "TADs".

**Response:** Thank you for all the specific comments. We revised all the mistakes mentioned here across the manuscript.

- Page 4, line 21: Reference numbering after 11 is shifted and need to be fixed. In addition, reference 11 is not related to TADs.

**Response:** Thank you for pointing out this mistake, which is corrected in the revised manuscript.

- Page 4, line 21: It would be appropriate to cite the study of Dong et al. (https://doi.org/10.1111/jipb.12809 )

**Response:** Thank you for pointing this out. We referenced and discussed the results presented in Dong *et al.* in Introduction (page 6, line 23-25 and page 7, line 18).

- Page 5, line 6-7: In addition of genome size, I would mention the chromosome size (500-800 Mbp) that is several times larger than maize and rice and may influence the folding domains.

**Response:** This is a good point, which we added to the revised manuscript (page 5, line 6 and page 7, line 5-6).

- Page 6, line 6-9: the origin of the introgression (rye) should me mentioned.

**Response:** Thank you for pointing this out. We added the origin of the introgression (page 5, line 24 - page 6, line 10).

- Page 7, line 3: "stringent read filtering" is a very generic term in this context, since the stringency of pairs filtering cannot be modified in HiC-Pro. However, the stringency of mapping can be tuned. If non-default setting were used, they should be fully described in the Method section. If not, "stringent" need to be deleted.

**Response:** Thank you for pointing this out. We used the default settings which is relatively stringent. In the revised manuscript, we added more detailed description, analyses and discussion about the mapping and filtering stringency in Results and Methods section (page 8, line 10 and the first one part of the Results section).

- Page 7, line 4: "map of genome packing" is commonly referred as "interaction map".

**Response:** Thank you for pointing this out. We revised "map of genome packing" to "interaction map" (page 8, line 13).

- Page 7, line 17: "with 10 kb bins" should be "at 10-kb resolution".

**Response:** Thank you for pointing this out. We revised the manuscript accordingly (page 12, line 13).

- Page 7, line 17: It should be clarified that the lack of "bias" refers to absence of differences of TADs density between the three sub-genomes.

**Response:** Thank you for pointing this out. We revised the description to “The abundance of TAD-like domains was similar across the three subgenomes ”. (page 12, line 16-17).

- Page 8, line 2: Reference 4 is related to bacteria and not relevant here.

**Response:** Thank you for pointing this out. We removed the reference.

- Page 10, line 1: Is the word "significantly" supported by any statistical test?

**Response:** Thank you for pointing this out. We added Wilcoxon T test，p<2e-16 (Fig. 6c legend).

- Page 10, line 18-21 and figure 5 a-b: The boxplot show the number of significant interactions, not the density of interactions (normalized by chromosome size and number). Does this calculation take in account A) the different chromosome size and B) the different number of possible interactions for each type (21 intra-chromosomal, 21 inter-chromosomal between homeologous, 63 within each sub-genome and 126 inter-chromosomal between different genomes) ?

**Response:** Thank you for pointing this out and sorry for the confusion. In the original version, we normalized the density of interaction by the number of possible interactions for each type, which needs to be mentioned in the manuscript. In the revised version, to be consistent across the manuscript, for showing the density of interactions, we displayed the number of inter-chromosome interactions normalized by the length of chromosomes (Figs. 1b,1d, 2a, 2b).

- The resolution of 5-kb for inter-chromosomal interactions seems unrealistic at this sequencing depth (Methods, page 15 line 16: "-res 5000 -window 25000 -minDist 10000000"). Moreover, a minimum distance of 10 Mega-bases does not exclude long-range intra-chromosomal interaction, considering the chromosome size in wheat is between 500 and 800 Mbp.

**Response:** Thank you for pointing this out. As mentioned in the responses to the major comments 3 and 4, we examined the distribution of fragment digested and chose 10 kb as resolution for all the analysis in the revised manuscript.

- Page 12 line 11-15: The existence of homology-mediated inter-chromosomal interactions is a major finding and should be placed among results rather than in Discussion. A finer analysis that takes in account the interactions between different sub-genomes, similarly to what done for Figure 5 c, would better support the hypothesis that the increased interactions between subgenomes A and B (Figure 5) is driven by sequence homology.

**Response:** We performed the analysis following the suggestion and obtained interesting results. We now put the findings to the Results section of manuscript. We revealed that the high within-subgenome inter-chromosome interaction is likely mediated by subgenome dominant homologous TEs, whereas the high AB inter-chromosome interaction is likely mediated by D suppressed TEs, namely, higher abundance in A and B. Please refer to the first two parts of the Result section and Figures 1-2.

- Page 17 line 20: Gene expression is measured as "RPM". To compare different genes within a given sample, it is necessary to normalize for the transcripts length (i.e. TPM or RPKM), as longer transcripts will generate more reads. Do RPM account for this factor? Which pipeline/software was used to analyze RNA-Seq data?
**Response:** Thank you for pointing this out. For measuring gene expression level, we used FPKM. We added the description to the Methods section (page 22, line 3-6). The y-axis of genome browser displaying RNA-seq data is normalized by RPM, which represents the coverage for each base pair, not each gene. In the revised manuscript, instead of single gene illustration, we plotted the genome-scale pattern correlating gene loop number and expression level (Fig. 6d). The illustration of *GW2* loops is removed since the *GW2* triads were connected by loops of ~2-3 Mb, which are not present in the loop list since the parameters were adjusted for detecting local loops only. In addition, there is an inversion in subgenome D. Despite that similar phenomenon was observed in Chinese Spring, we can’t conclude whether the lower expression of *GW2-D* is due to the inversion or reduced loop number or both.

Figures and legends:
- Legend of figure 1c: "fractions of genes" is unclear. Does this refer to average gene coverage? How the coverage was calculated? Sliding window, non-overlapping bins? In either case, of which size?

**Response:** Sorry for the confusion. We revised the figure legend. Figure 1c (Fig. 4c in the current version) is the cumulative distribution of TAD size. The x-axis is the TAD length, and the y-axis is the fraction of TADs whose size is smaller than given value on x-axis.

- Legend of Figure 1d: when referring to the density of genomic features (genes, TEs, smRNAs, etc) the unit of measure should be specified on the Y-axis (%, genes/Mb, etc.)

**Response:** Thank you for pointing this out. We revised the labels of y-axis in Figures 4d, 4f, and 5d.

- Legend of Figure 1e: "Distribution of expression density of genes" is very unclear. Is it possibly "distribution of gene expression levels"? I suggest to use a linear scale instead of log2 transformed values and/or exclude the outliers to improve the readability of this chart.

**Response:** Thank you for the suggestion, we made the revisions accordingly. Please refer to Figure 4e in the revised manuscript.

- Page 8, line 10: "Fig 2" and "Fig 3" are mislabeled throughout the text. According to the order in which they are mentioned in the text, Figure 3, that shows motifs, should go before figure 2, that illustrates DNA methylation.

**Response:** Sorry for the confusion. We corrected the order, which are Figure 4f and Figure 5 in the revised version.

- Legend of Figure 4d: scale on the X-axis is missing.

**Response:** Thank you. In the revised manuscript, instead of single gene illustration, we plotted the genome-scale pattern correlating variant loop number and gene expression (Fig. 6d in the revised manuscript).

- The interaction intensity between for different type of chromosomes is measured as the number of significant interactions (Figure 5a-b), while the interaction intensity between different sub-genomes (Figure 5 c), between the introgressed chromosome arm 1RS and the rest of the genome (Figure 6 b) is measured as interaction density. What is the reason for using different measures? Is it possible to use a single measure for all the interaction intensities?

**Response:** Thank you for the suggestion. In the revised manuscript, we used a single measure (the number of interactions) for showing the density of interactions. The contacts were normalized by chromosome lengths for chromosome interactions both within and between chromosomes (Fig. 1 and Fig. 2a,b). For 1B/1R interactions with the rest of the genome, the number of contacts within 10 Kb were calculated (Fig. 3b). For Circos plot displaying the interaction of 1B/1R with the rest of the genome, the significant interactions were shown (Fig. 3a), which is consistent with the number of contacts (Fig. 3b).

- Similarly, in Figure 5 a-b is plotted the number of significant interactions, but the level of sequence homology in Supplemental Figure 2 uses all pairwise interactions. Why different sets of pairwise interactions are used? How would the plots look by using the same set of interactions?

**Response:** Sorry for the confusion. Supplemental Figure 2 depicting the level of sequence homology in last version of the manuscript also used the number of significant interactions, which is modified and put to Figure 2c-d in the revised manuscript.

**Reviewer2：**

**1)**	It seems that all sequencing libraries and primary sequencing data of Hi-C, RNA-seq and BS-seq in this study were prepared and generated by a third-party profit company Novogene. What I am really concerned is the data quality controls, which were not described in the manuscript including the replicate information, reproducibility between replicates if they have. In addition, the ratio of valid reads in Hi-C assay is too low that thus reduce the reliability of conclusions based on it.

**Response:** Thank you for pointing this out. We added the detailed statistics for all sequencing data (Supplemental Table 1). The relatively low Hi-C ratio is because the default settings of HiCPro is stringent. We investigated the impact of mapping and filtering stringencies on the result in the revised manuscript (the first two parts of the Result section and Figures 1-2). Here is a summary also including another two publically available hexaploid wheat Hi-C data. Our data have relatively high sequencing depth and mapping statistics.

**2)**	They identified tens of thousands of TADs and similar number of chromatin loops, however, they observed that the median size of common TADs (~200 kb) is smaller than the loops (400 kb). We usually consider that chromatin loops are kind of sub-TADs features and have the shorter genomic spans.

**Response:** Thank you for pointing this out. The length of loops may have a wide range, the detection of which is dependent on the procedures and parameters. We followed similar procedure for loop detection with this human study (doi.org/10.1016/j.cell.2014.11.021), in which the median length of TADs and loops are 185 and 275 kb, respectively.

**3)**	They described in the main text that they try to detect the chromatin loops following the method of Eres's 2014 Cell paper (Rao et al). However, they stated in the Method section that the TADs and Loops were called using a suite of Homer software "findTADsAndLoops.pl" published in 2010. I am confused with the inconsistent analysis methods.

**Response:** Thank you for pointing this out and sorry for the confusion. We removed the citation of this Cell paper in the revised manuscript since it was cited to describe the principle for loop detection. For TAD and loop definition, we used the the updated script "findTADsAndLoops.pl" implemented in HOMER. We added the description in page 19, line 19, and updated the reference to DOI: 10.1016/j.cell.2018.07.047, as recommended by the Homer website:

**4)**	The authors characterized the epigenomic features of TAD boundaries using DNA methylation data, but they failed to provide any novel conclusion. In addition, the same group had published a bunch of wheat histone modifications data (Li et al., Genome Biology 2019). I would suggest the authors take a look at the epigenomic features of TAD boundaries with these datasets.

**Response:** Thank you for the suggestion. The Hi-C data is from a wheat cultivar AK58 with 1B/1R translocation, but the epigenomic data were generated in Chinese Spring. The major topic for this version of the manuscript is the interplay between sequence context and higher-order chromatin sturcture. We are generating AK58 epigenomic data, and hope to correlate the epigenetic marks and higher-order maps in the near future.

**5)**	I suggest the authors to do some validations for detected high-frequent interacting anchors using FISH or 3C methods.

**Response:** Thank you for the suggestion. The loops were validated by the recently published 3C-PCR results that were used to test the Hi-C data for CS wheat (doi.org/10.1186/s13059-020-01998-1). All four CS loops had co-linear regions in AK58, and three were also detected as loops in AK58, whereas the fourth was localized to the TAD boundaries in AK58 (Supplemental Fig. 6)(please also refer to page 20, line 13-17).

**6)** The Discussion is too perfunctory since many plant 3D genome studies had been published and the authors did not cite any of them in this part of the manuscript.

**Response:** Thank you for pointing this out. We re-wrote the discussion section. Please refer to page 14-17.

**7)**	This manuscript was prepared in a hurry and a lot of writing issues including incorrect citation (e.g. reference #11 in p4-line21), Font inconsistence (e.g. p14-line2) and format issues (e.g. p16-line14) could be easily found throughout the paragraphs.

**Response:** We apologize for the haste of the last version of manuscript, which we carefully revised according to all the comments.

**Second round of review**

**Reviewer 2**

The authors addressed some of my initial the comments and the revised manuscript is now much better. There are some minor issues that need to be modified.

My specific comments:
1.     P3-line10, “inter-chromosomal interact” should be “inter-chromosomal interactions”.
2.     P3-line19, the conclusion “The chromatin loops were mostly localized to TAD boundaries” is based on the results in page 14 (line 11) “Moreover, 74.3% of the local anchored pairs were located within or surrounding TAD-like domains, and about half (65.9%) were localized within gene bodies or promoter regions (3 kb upstream of a transcription start site)” and Figure 6a; As TAD boundaries and TAD-like domains are different genomic regions, I think “the chromatin loops were localized around TAD boundaries” should be more precise. Another thing, have no idea about the sentence of p14-line11 refers to which figures?
3.     P6-lines 6–9, references?
4.     P7-lines 3–5, the most recent study on TCP protein in 3D genome architecture (Ezgi Süheyla Karaaslan et. al., 2020, Nat Plants) need to be cited.
5.     P7-line11, Zheng’s paper is a nice review on 3D genome, but it is not an appropriate reference here, because “loop extrusion” is not the major topic of this paper, and there are some original publications on “loop extrusion” not to be cited here. Same issue for the citation on page 7 (line 17), for rice chromatin loops, I think the paper by Zhao’s Nature communications paper (2019,10:3640) is a better one for your reference.
6.     P12-lines 3–7, “... possibly because of the relatively low sequence homology between 1RS and wheat chromosomes”, is there any difference of TE density, CHH context, and gene expression level between the alien 1RS and its recipient genome? If possible, please construct Hi-C maps of the rye and compare the interchromosomal interactions between the 1RS and other rye chromosomes.
7.     P12-line14, it seems that Fig. 4c does not correspond to this sentence, but to the next sentence (lines 18–19) “... 220 kb”.
8.     P14-lines 15–17, “close to the anchor (< 3 kb)” is inconsistent with Fig 6c (with/without loop as showed in Fig. 6c).
9.     P15-line24 and p16-lines 1–7, “... a major driving force of chromosome folding is likely derived from the centromere. The high density of TEs surrounding centromeres and inter-chromosomal interactions within subgenomes may facilitate the formation of the Rabl structure”. Someone may argue that different types of cells from the same species have identical sequence context but adopt different chromosome configurations. For example, As reviewed by Ezgi Süheyla Doğan and Chang Liu (Nat Plants, 2018), rice chromosomes in xylem vessel cell nuclei show a “Rabl” conformation, whereas chromosomes from rice leaf cells display a “non-Rabl” arrangement, and chromosomes of meiotic rice cells exhibit a “Bouquet” configuration. I therefore disagree with your comments and please revise it.
10.     Some sentences are too complicated to understand, which need to be polished by the authors. For example, p5-line24.

**Authors Response**

We sincerely thank the reviewer for the cogent comments to help us improve our manuscript, which we have addressed in detail below.

Reviewer #2: The authors addressed some of my initial the comments and the revised manuscript is now much better. There are some minor issues that need to be modified.

My specific comments:

1. P3-line10, “inter-chromosomal interact” should be “inter-chromosomal interactions”.

**Response:** We thank the reviewer for pointing this out, which we have corrected in the revised manuscript (P3-line9).

2. P3-line19, the conclusion “The chromatin loops were mostly localized to TAD boundaries” is based on the results in page 14 (line 11) “Moreover, 74.3% of the local anchored pairs were located within or surrounding TAD-like domains, and about half (65.9%) were localized within gene bodies or promoter regions (3 kb upstream of a transcription start site)” and Figure 6a; As TAD boundaries and TAD-like domains are different genomic regions, I think “the chromatin loops were localized around TAD boundaries” should be more precise. Another thing, have no idea about the sentence of p14-line11 refers to which figures?

**Response:** We thank the reviewer for pointing this out. We revised the sentence in the abstract (P3-line18) to “The chromatin loops were mostly localized around TAD boundaries”. In addition, p14-line 11 was description of the distribution without figure. We now provide Supplemental Figure 3 in the revised version corresponding to the description.

3. P6-lines 6–9, references?

**Response:** Aikang 58 is the major founder parent for wheat breeding in China and has won the first-prize of National Science and Technology Progress Award, but there was no academic paper specifically describing its breeding progress. Here we added a reference demonstrating the superiority in height and resistance compare to other popular wheat cultivars. Please refer to P6-line 6.

4. P7-lines 3–5, the most recent study on TCP protein in 3D genome architecture (Ezgi Süheyla Karaaslan et. al., 2020, Nat Plants) need to be cited.

**Response:** We cited the reference in P7-line2.

5. P7-line11, Zheng’s paper is a nice review on 3D genome, but it is not an appropriate reference here, because “loop extrusion” is not the major topic of this paper, and there are some original publications on “loop extrusion” not to be cited here. Same issue for the citation on page 7 (line 17), for rice chromatin loops, I think the paper by Zhao’s Nature communications paper (2019,10:3640) is a better one for your reference.

**Response:** We thank the reviewer for pointing these out. In the revised manuscript, we used the first experimental evidence supporting “loop extrusion” as the reference (P7-line8 and P13-line 23). For rice chromatin loops, we used the recommended reference (P7-line13).

6. P12-lines 3–7, “... possibly because of the relatively low sequence homology between 1RS and wheat chromosomes”, is there any difference of TE density, CHH context, and gene expression level between the alien 1RS and its recipient genome? If possible, please construct Hi-C maps of the rye and compare the interchromosomal interactions between the 1RS and other rye chromosomes.

**Response:** We thank the reviewer for this suggestion. We observed low sequence homology, different TE subfamily density, as well as different CHH methylation level between 1RS and the recipient genome (Fig. 3e-h). These findings enriched the results of introgression part.

Regarding the rye Hi-C map, the project is led by Prof. Daowen Wang, who kindly shared their Hi-C data with us. Here is the Hi-C map in rye, where the interaction frequency between 1RS and other chromosomes is comparable as compared to the inter-chromosomal interactions between other chromosomes.

**rye**

**AK58 subgenome B**

The left figure is in the rye genome sequencing manuscript, which will be online soon.

7. P12-line14, it seems that Fig. 4c does not correspond to this sentence, but to the next sentence (lines 18–19) “... 220 kb”.

**Response:** We thank the reviewer for pointing this out. In the revised version, the reference to Fig. 4c has been moved to P12-line15 after “… 220Kb”.

8. P14-lines 15–17, “close to the anchor (< 3 kb)” is inconsistent with Fig 6c (with/without loop as showed in Fig. 6c).

**Response:** We thank the reviewer for pointing this out. We revised the figure label to with/without loop < 3 kb. Please refer to Fig. 6c.

9. P15-line24 and p16-lines 1–7, “... a major driving force of chromosome folding is likely derived from the centromere. The high density of TEs surrounding centromeres and inter-chromosomal interactions within subgenomes may facilitate the formation of the Rabl structure”. Someone may argue that different types of cells from the same species have identical sequence context but adopt different chromosome configurations. For example, As reviewed by Ezgi Süheyla Doğan and Chang Liu (Nat Plants, 2018), rice chromosomes in xylem vessel cell nuclei show a “Rabl” conformation, whereas chromosomes from rice leaf cells display a “non-Rabl” arrangement, and chromosomes of meiotic rice cells exhibit a “Bouquet” configuration. I therefore disagree with your comments and please revise it.

**Response:** We thank the reviewer for all these information. We added the specific restriction to the statement “implying in wheat cells with Rabl configuration, a major driving force of chromosome folding is likely derived from the centromere.” Please refer to P15-line17.

10. Some sentences are too complicated to understand, which need to be polished by the authors. For example, p5-line24.

**Response:** We thank the reviewer for pointing this out. We re-wrote the sentence to be clearer (P5-line22). We also checked throughout the manuscript to ensure that there is no sentence too long or too complicated.
