# Supplementary material for: Author Correction: Homology-mediated inter-chromosomal interactions in hexaploid wheat lead to specific subgenome territories following polyploidization and introgression
Source: Genome Biol. 2024 Jan 2;25:5. doi: 10.1186/s13059-023-03154-x (PMC10763112; doi:10.1186/s13059-023-03154-x)
Supplement: Supplementary file 1 — Additional file 4. Additional analyses. [file 13059_2023_3154_MOESM1_ESM.zip › Hi-C_supplementary_analysis 1212v2.docx]

In the published research [1], we found that the within-subgenome interactions were more pronounced when the multi-mapped read pairs were included (Fig2c-f). Further analysis revealed that subgenome dominant transposable elements (TEs) were closely associated with regions mediating within-subgenome interactions (Fig 2g-h).

Here, as a supplement, for within and between subgenome interactions, we quantitatively compared the relative abundance of different types of subgenome biased TEs in anchor pairs with high sequence similarity, and calculated their fold enrichment relative to balanced TEs (scripts provided). The quantitative result demonstrated clear association between subgenome-dominant TEs and within subgenome interactions, regardless of with or without multi-mapped read, demonstrating that the conclusion is robust.

Similar results were obtained when using Hi-C data from Chinese Spring (bottom panels) (doi:10.1186/s13059-020-01998-1.).

The code could be run in Linux by “bash inter_chr_interaction_TE_pattern.sh”.
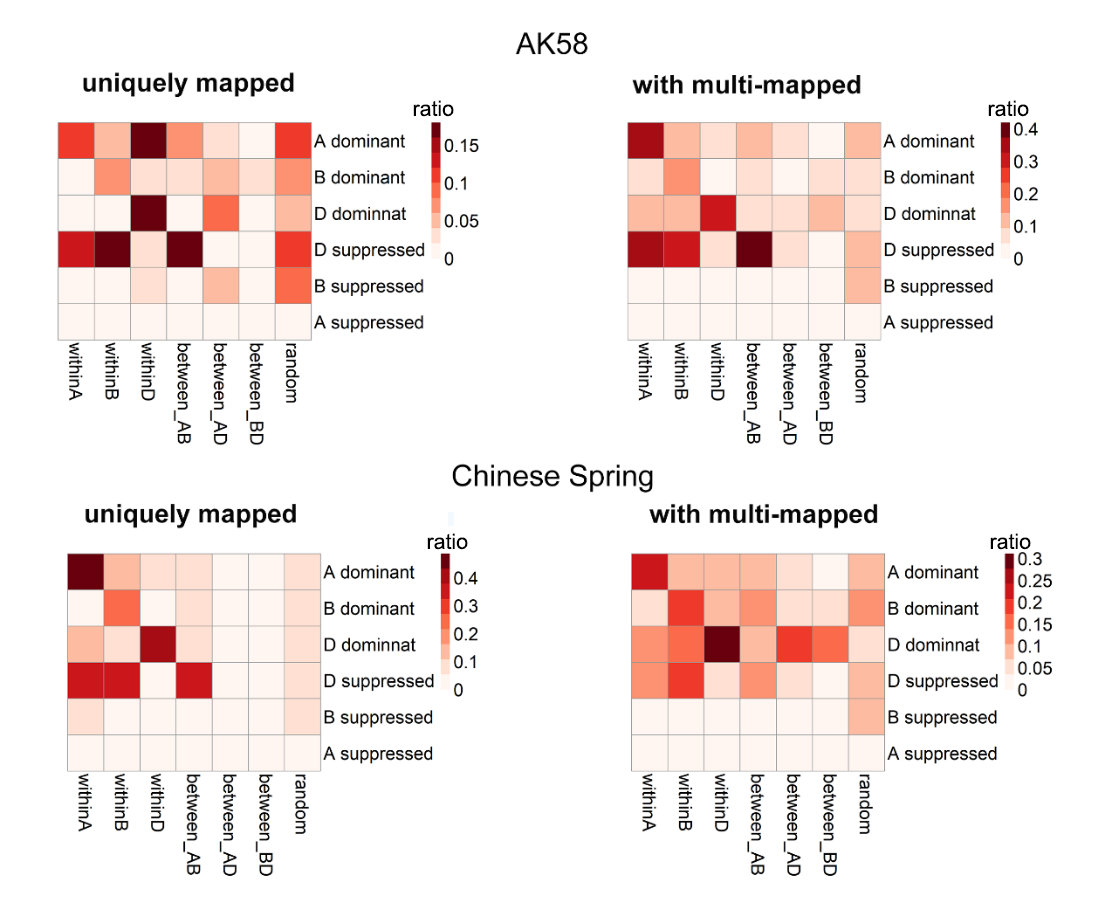


**Legend:** As a supplement to Figure 2h, we calculated quantitative association between subgenome biased TEs (each role) and regions mediating inter-chromosomal interaction between or within subgenome(s) (each column) in AK58 (upper panels) and Chinese Spring (bottom panels). For Hi-C data mapping, uniquely mapped read (left panels) or with multi-mapped read (right panels) were used. The relative abundance of different types of subgenome biased TEs in anchor pairs with high sequence similarity were quantitatively compared, and their fold change (**ratio**) relative to balanced TEs were calculated.

1. Jia JZ, Xie YL, Cheng JF, Kong CZ, Wang MY, Gao LF, Zhao F, Guo JY, Wang K, Li GW, et al: **Homology-mediated inter-chromosomal interactions in hexaploid wheat lead to specific subgenome territories following polyploidization and introgression.** *Genome Biology* 2021, **22**.
